# Characteristics and properties of nano-LiCoO_2_ synthesized by pre-organized single source precursors: Li-ion diffusivity, electrochemistry and biological assessment

**DOI:** 10.1186/s12951-017-0292-3

**Published:** 2017-08-22

**Authors:** Jean-Pierre Brog, Aurélien Crochet, Joël Seydoux, Martin J. D. Clift, Benoît Baichette, Sivarajakumar Maharajan, Hana Barosova, Pierre Brodard, Mariana Spodaryk, Andreas Züttel, Barbara Rothen-Rutishauser, Nam Hee Kwon, Katharina M. Fromm

**Affiliations:** 1 Department of Chemistry, University of Fribourg, Chemin du Musée 9, 1700 Fribourg, Switzerland; 2 Fribourg Center for Nanomaterials FriMat, University of Fribourg, Chemin du Musée 9, 1700 Fribourg, Switzerland; 3 Adolphe Merkle Institute, University of Fribourg, 1700 Fribourg, Switzerland; 4 College of Engineering and Architecture of Fribourg, University of Applied Sciences of Western Switzerland, Boulevard de Pérolles 80, 1705 Fribourg, Switzerland; 5 Laboratory of Materials for Renewable Energy (LMER), ISIC-SB, École Polytechnique Fédérale de Lausanne (EPFL), Valais/Wallis Energypolis, Rue de l’Industrie 17, 1951 Sion, Switzerland

**Keywords:** Single source precursors, Nano-LiCoO_2_, Li^+^ Diffusion coefficient, Li-ion batteries, Nanoparticle hazard

## Abstract

**Background:**

LiCoO_2_ is one of the most used cathode materials in Li-ion batteries. Its conventional synthesis requires high temperature (>800 °C) and long heating time (>24 h) to obtain the micronscale rhombohedral layered high-temperature phase of LiCoO_2_ (HT-LCO). Nanoscale HT-LCO is of interest to improve the battery performance as the lithium (Li^+^) ion pathway is expected to be shorter in nanoparticles as compared to micron sized ones. Since batteries typically get recycled, the exposure to nanoparticles during this process needs to be evaluated.

**Results:**

Several new single source precursors containing lithium (Li^+^) and cobalt (Co^2+^) ions, based on alkoxides and aryloxides have been structurally characterized and were thermally transformed into nanoscale HT-LCO at 450 °C within few hours. The size of the nanoparticles depends on the precursor, determining the electrochemical performance. The Li-ion diffusion coefficients of our LiCoO_2_ nanoparticles improved at least by a factor of 10 compared to commercial one, while showing good reversibility upon charging and discharging. The hazard of occupational exposure to nanoparticles during battery recycling was investigated with an in vitro multicellular lung model.

**Conclusions:**

Our heterobimetallic single source precursors allow to dramatically reduce the production temperature and time for HT-LCO. The obtained nanoparticles of LiCoO_2_ have faster kinetics for Li^+^ insertion/extraction compared to microparticles. Overall, nano-sized LiCoO_2_ particles indicate a lower cytotoxic and (*pro*-)inflammogenic potential in vitro compared to their micron-sized counterparts. However, nanoparticles aggregate in air and behave partially like microparticles.

**Electronic supplementary material:**

The online version of this article (doi:10.1186/s12951-017-0292-3) contains supplementary material, which is available to authorized users.

## Background

Lithium cobalt oxide LiCoO_2_ has been the most commonly used cathode material in rechargeable Li-ion batteries since Goodenough first introduced the reversible reaction of Li-ions in the structure [1]. The structures of Li_1−x_CoO_2_ have been extensively studied as a function of lithium de-intercalation, leading to several phase transformations from rhombohedral with 0.06 < x < 0.25 [2–5], via monoclinic with x = 0.5 [2, 3], to hexagonal for 0.66 < x < 0.83 [6, 7], and a second hexagonal phase, O1, for 0.88 < x < 1 [6–8].

The layered structure of lithiated LiCoO_2_ exhibits two crystal structures depending on the temperature during synthesis and the preparation method. LiCoO_2_ produced at low temperature (~400 °C) (LT-LCO) has a cubic spinel structure with the space group *F*d3 m [9, 10] while the phase synthesized at high temperature (>850 °C, HT-LCO) has a rhombohedral layered structure [11]. LT-LCO shows a large hysteresis between the intercalation and de-intercalation of lithium ions [12–14], which is due to the mixing of Co^3+^ and Li^+^ in the structure, preventing the formation of layered pathways for Li-ion diffusion. The material is therefore calcined at higher temperature to yield HT-LCO, which possesses alternating planes of Co^3+^ and Li^+^ cations in the hexagonal ABCABC oxygen packing [15], providing superior electrochemical properties in Li-ion batteries [16].

Industrially, two starting materials, typically Li_2_CO_3_ and Co_3_O_4_, are heated in a two-step process to yield first at a temperature of <600 °C for 24 h under O_2_ the LT-LCO. A second calcination step at 900 °C for >12 h under O_2_ [17] yields the HT-LCO [18–20]. Such a prolonged calcination process at high temperature causes however coarsening of the particles and evaporation of lithium [21]. Various synthetic methods have thus been investigated to avoid the high temperature process, with the aim to obtain the rhombohedral layered structure of HT-LCO, e.g. sol–gel [22–25], hydrothermal [26], or precipitation [16]. However, low temperature syntheses formed mostly the cubic spinel LT-LiCoO_2_, which is not favorable for Li^+^ insertion/extraction. Thus, calcination at high temperature >800 °C was always required in a second step to use the so-produced material in Li-ion battery cathodes [16].

Another access to the layered structure of HT-LCO uses metal–organic single source precursors based on alkoxides or aryloxides, in which the metal ions are already preorganized. Indeed, the synthesis of heterobimetallic alkoxides and/or aryloxides can provide a facile route for obtaining soluble, volatile, and generally monomeric species, that can thus serve as valuable precursors for making metal oxides under rather mild conditions [27–36]. For example, Buzzeo published homoleptic cobalt phenolate compounds of the type K_2_[Co(OAr)_4_] (OAr = OC_6_F_5_^−^ or 3,5-OC_6_H_3_(CF_3_)_2_^−^), in which the effect of fluorination of phenoxide on (K18C6)_2_[Co(OAr)_4_] is highlighted [37]. Boyle et al. published lithium cobalt double aryloxide compounds obtained from LiN(SiMe_3_)_2_, Co(N(SiMe_3_)_2_)_2_ in THF and subsequent addition of an aryl alcohol. They obtained nanoparticles of LiCoO_2_ by thin film formation [38], but did not characterize them electrochemically. Nanoparticles of HT-LCO have the advantage to offer shorter diffusion lengths for the Li-ions as compared to the commercial, micron-sized particles from which only ~50% of Li-ions can be used [26, 35]. On the other hand, since batteries are typically also shredded upon recycling, the use of nanomaterials in batteries might present a certain danger, which requires a risk management for new materials.

In this context, we present here new molecular precursors using simple ligands such as phenoxide and alkoxides with a low amount of carbon atoms that can produce nano-HT-LCO at quite low temperature. We have tested the new materials for their electrochemical properties in cathodes and their Li-ion diffusion coefficients were determined. In order to evaluate possible material hazards, the nanoparticles of HT-LCO were exposed directly at the air–liquid interface (ALI) using a well-established in vitro multicellular lung model [39]. The lung was chosen as an experimental tissue, since it can be considered by far the most important portal of entry for aerosolized nanoparticles into the human body [40–46]. Although various aspects of nanoparticles toxicity have already been described and studied in the recent literature, almost no studies were carried out in the domain of battery cathode nanoparticles.

## Methods

### Materials and reagents

Cobalt chloride (CoCl_2_) (dry or hydrated with two H_2_O), lithium phenoxide (LiOPh) in tetrahydrofuran (THF), lithium *iso*-propoxide (LiO^*i*^Pr) in THF, ethanol (technical grade and analytical grade), tetramethylethylenediamine **(**TMEDA), dioxane, dimethoxyethane (DME), pyridine (Py), heptane and micron-sized HT-LiCoO_2_ were purchased from Sigma-Aldrich (Switzerland). Lithium *tert*-butoxide (LiO^*t*^Bu) in THF, lithium methoxide (LiOMe) in methanol, lithium ethoxide (LiOEt) in THF and THF (dry and over molecular sieves) were purchased from Acros Organics (Belgium). Deionized water was produced in house by double distillation.

### Synthesis of bimetallic complexes [47]

All experiments were carried out under an inert argon atmosphere, using Schlenk techniques [48]. All solvents were bought dried and were stored over molecular sieve. The elemental analysis of the compounds turned out to be difficult to obtain due to the instability of most compounds in air, based on the loss of (coordinated) solvent.

The compounds [Co(OPh)_4_Li_2_(THF)_4_] (**1**), [Co(OPh)_4_Li_2_(THF)_4_]·THF (**2**), [Co(OPh)_4_Li_2_(THF)_2_(H_2_O)(THF)_2_]_2_ (**3**), [Co(OPh)_4_Li_2_(TMEDA)_2_] (**4**), [Co(OPh)_4_Li_2_(dioxane)_2_]_n_ (**5**), [Co(OPh)_4_Li_2_(DME)_2_] (**6**), [Co(OPh)_4_Li_2_(Py)_4_] (**7**), [Co_2_(O^*t*^Bu)_6_Li_4_(THF)_2_] (**8**), [Co_2_(O^*t*^Bu)_2_(OPh)_4_Li_2_(THF)_4_] (**9**), [Co_2_(O^*i*^Pr)_6_Li_2_(THF)_2_] (**10**), [Co_2_(OEt)_12_Li_8_(THF)_8–10_] (**11**), and [Co_2_(OMe)_6_Li_2_(THF)_2_(MeOH)_2_] (**12**) were synthesized using CoCl_2_ as starting material and reacting it with the corresponding lithium aryloxide or alkoxide. In a typical reaction procedure, dried CoCl_2_ is dissolved in dry THF under heating to reflux. After stirring for 30 min, aliquots of LiOR (R = Ph, ^*t*^Bu, ^*i*^Pr, Et, Me) are added. The mixture is heated to reflux, stirred during 30 min and then concentrated. Layering the concentrated solution with a non-solvent, respectively solvent exchange lead to single crystalline material for compounds **1**–**5** and **9**, while powders were obtained for **6**–**8** and **10**–**12**. Table 1 resumes the reaction conditions for all compounds, with detailed synthesis protocols and IR-analyses given in the Additional file 1: Text 1.

**Table 1:** The reactants, synthetic conditions and the yields of the compounds **1**, **8**-**12**

Compound	Formula	Reactants in synthesis	Yields (%)
**1**	[Co(OPh)_4_Li_2_(THF)_4_]	CoCl_2_ (0.1 g, 0.77 mmol) + 4 LiOPh 1 M in THF (3.1 ml, 3.1 mmol)	82
**2**	[Co(OPh)_4_Li_2_(THF)_4_]·THF	Idem as **1**, but −24 °C under argon	56
**3**	[Co(OPh)_4_Li_2_(THF)_2_(H_2_O)(THF)_2_]_2_	Idem as **1**, but −24 °C in air	<10
**4**	[Co(OPh)_4_Li_2_(TMEDA)_2_]	Idem as **1**, recrystallized from TMEDA	69
**5**	[Co(OPh)_4_Li_2_(dioxane)_2_]_n_	Idem as **1**, recrystallized from dioxane	95
**6**	[Co(OPh)_4_Li_2_(DME)_2_]	Idem as **1**, recrystallized from DME	47
**7**	[Co(OPh)_4_Li_2_(Py)_4_]	Idem as **1**, recrystallized from pyridine	39
**8**	[Co_2_(O^*t*^Bu)_6_Li_4_(THF)_2_]	CoCl_2_ (585 mg, 4.5 mmol) + 3 LiO^*t*^Bu 1 M in THF 13.5 ml (13.5 mmol)	87
**9**	[Co_2_(O^*t*^Bu)_2_(OPh)_4_Li_2_(THF)_4_]	CoCl_2_ (500 mg, 3.85 mmol) + LiO^*t*^Bu (3.9 ml, 3.9 mmol) + LiOPh 1 M in THF (7.7 ml, 7.7 mmol)	85
**10**	[Co_2_(O^*i*^Pr)_6_Li_2_(THF)_2_]	CoCl_2_ (500 mg, 3.85 mmol) + 3 LiO^*i*^Pr 2 M in THF (5.8 ml, 11.6 mmol)	92
**11**	[Co_2_(OEt)_12_Li_8_ (THF)_8-10_]	CoCl_2_ (500 mg, 3.85 mmol) + 6 LiOEt 2 M in THF (11.6 ml, 23.2 mmol)	89
**12**	[Co_2_(OMe)_6_Li_2_(THF)_2_(MeOH)_2_]	CoCl_2_ (500 mg, 3.85 mmol) + 3 LiOMe 2 M in THF (5.3 ml, 11.7 mmol) and MeOH	90

### Calcination to LiCoO_2_

Among the so obtained precursors, compounds **1, 8**–**12** were heated up to 450 °C for 1 h and 500 °C for 2 h at an average rate of 18 °C/min under an air flow of 8 l/min in a muffle furnace equipped with an evacuation smokestack for combustion gases. The black powder obtained was then cooled to room temperature within 5 min in air. The black/grey powder was next washed by centrifugation three times with water and two times with ethanol in order to remove LiCl. The clean and dry oxide nanopowder was finally annealed using an average ramp of 17 °C/min up to 600 °C for 80 min to remove low temperature oxide phase impurities. These materials were used for the biohazard tests. LiCoO_2_ prepared with LiOMe and LiO^*t*^Bu was calcined further until 700 °C for 30 min to measure charge/discharge capacities at different current densities.

### Characterization

#### Single crystal X-ray structures

Single crystals of compounds **1**–**5** and **9** were mounted on a loop and all geometric and intensity data were taken from these crystals. Data collection using Mo-K_α1_ radiation (λ = 0.71073 Å) was performed at 150 K on a STOE IPDS-II diffractometer equipped with an Oxford Cryosystem open flow cryostat [49]. Absorption correction was partially integrated in the data reduction procedure [50]. The structure was solved by SIR 2004 and refined using full-matrix least-squares on *F* ^*2*^ with the SHELX-97 package [51, 52]. All heavy atoms could be refined anisotropically. Hydrogen atoms were introduced as fixed contributors when a residual electronic density was observed near their expected positions. Diffraction data sets for compounds **1**–**5** are unfortunately incomplete due to decomposition of the single crystals, resulting in poor data sets and *R*-values for the compounds. However, the isotropic attribution of heavy atoms is unambiguous.

Crystallographic data (excluding structure factors) for the structures in this paper have been deposited with the Cambridge Crystallographic Data Center, 12 Union Road, Cambridge CB21EZ, UK. Copies of the data can be obtained on quoting the depositing numbers CCDC- 1527018 (**1**), 1527022 (**2**), 1527023 (**3**), 1527020 (**4**), 1527019 (**5**), and 1527021 (**9**) (Fax: +44-1223-336-033; E-mail: deposit@ccdc.cam.ac.uk). Important crystal data for these compounds are given in the Additional file 1: Table S1.

#### Other characterizations

For powder XRD measurements, a Stoe IPDS II theta, equipped with monochromated Mo-K_α1_ radiation (0.71073 Å) was used in order to avoid X-ray fluorescence of the cobalt but also a Stoe STADIP, equipped with monochromated Cu-K_α1_ radiation (1.540598 Å) and Mythen detector. TGA was recorded on a Mettler Toledo TGA/SDTA851e in closed aluminium crucibles with a pin hole. Specific surface area was measured on a Micromeritics Gemini V series BET with a pre-treatment under vacuum at 150 °C for one night. SEM images were recorded on Phenom Desktop SEM and a FEI XL 30 Sirion FEG with Secondary Electron and EDS Energy Dispersive Spectrometer detectors. SEM samples were prepared by spraying them on a carbon tape glued on a SEM holder to reproduce the spraying in the exposure chamber. All images were obtained without sputter coating pretreatment. TEM images were recorded on a FEI/Philips CM-100 Biotwin. Raman spectra were recorded with a confocal micro-Raman spectrometer, HORIBA LabRAM HR800, combined with an optical microscope Olympus BX41, using a red laser at 633 nm for excitation, attenuated with filters in order to avoid thermal degradation of the scotch tape used as sample holder. The Li^+^ and Co^2+/3+^ ion concentrations were determined by inductively coupled plasma optical emission spectroscopy (ICP-OES) using a Perkin Elmer Optima 7000DV.

The muffle furnace used for combustion and tempering is equipped with a eurotherm thermal controller (Tony Güller Naber Industrieofenbau, Zurich, Switzerland).

### Metal ion release

A metal ion release test was conducted to assess the amount of potential metal ion dissolution from the tested compounds. 100 mg of each of the micro- and nanoparticles were immersed in 10 ml of deionised water at pH 7 and pH 4.5 for 24 h. The concentrations of the metal ions were then determined using ICP measurements (Additional file 1).

### Statistical and data analysis

The microparticles of LiCoO_2_ are represented in black and the nanoparticles in grey bars. Data are the mean ± the standard error of the mean (SEM) and are absolute values. Values were considered significantly different compared to the negative control with p < 0.05 using a one way Anova with a post hoc Tukey test (*nanoparticles, #microparticles).

### Electrodes and electrochemical tests

#### Preparation of the electrodes

0.5 g of the nanoscale-LiCoO_2_ and 10 wt% SFG graphite with respect to LiCoO_2_ were ball milled in a horizontal set-up (Retch MM 400) for 15 min at a frequency of 30 Hz. The ball milling jar had a volume of 10 ml and contained two stainless steel balls of 10 mm in diameter. The electrode paste was prepared in a glass tube, starting with polyvinylidene fluoride (PVDF) (10 wt% with respect to LiCoO_2_) and 0.5 ml of N-methyl-2-pyrrolidone (NMP), which were stirred by a mechanical stirrer for 30 min until PVDF was completely dissolved. 2 wt% of ABG graphite with respect to LiCoO_2_ was then added and the mixture was stirred for 15 min. Then, the ball milled composite powder (0.6 g) of graphite and LiCoO_2_ were added to the PVDF/graphite/NMP mix and stirred for a half an hour. The so-obtained paste of PVDF/NMP/graphite/LiCoO_2_ was spread onto an aluminum foil by the doctor-blade method and dried overnight at 120 °C. The overall weight ratio of the composite made of nano-LiCoO_2_ (active material), carbon and binder was around 78:12:10.

#### Cell assembly

All compounds used were dried to avoid HF formation in the electrolyte and were assembled in a glove box under argon (MBraun, Germany) having <0.1 ppm of water and oxygen. Typically, the LiCoO_2_ electrode was assembled in a coin cell using lithium metal as anode, a few drops of an ethyl carbonate (EC) and diethylene carbonate (DEC) mixture in a 1:1 volume ratio with 1 M LiPF_6_ and 2 wt% of vinylene carbonate as electrolyte with respect to solvents and LiPF_6_ as well as a Celgard separator.

#### Battery tests

A potentiostat, Princeton Applied Research 273A, and an Arbin battery test instrument (version 4.27) were used to examine the electrochemical properties of the carbon-nano-LiCoO_2_ composite electrodes. Charge and discharge capacities of coin cells were measured by an Arbin 2000 battery test instrument at different current densities of C/20, C/10, C/5, C/2 and 1C. The voltage window was set between 2.6 and 4.4 V vs. Li^+^/Li. The current densities between C/20 and 1C were based on the practical capacity of 140 mAh/g.

Li-ion diffusion coefficients were evaluated by cyclic voltammetry at a sweep rate of 1, 0.7, 0.5, 0.2 and 0.1 mV/s between 3.5 and 4.4 V vs. Li^+^/Li.

The discharge kinetic of LiCoO_2_ electrodes was investigated at various current densities between 20C and C/20. The LiCoO_2_ coin cells were re-charged until 4.4 V vs. Li^+^/Li at 20C current density and then rested for 3 min. The electrode was discharged at the same current density of 20C until 2.6 V. This procedure was repeated at various lower current densities until C/20 (so-called deep discharge). By this procedure, the capacity vs. the discharge current can be determined directly. The sum of all capacities, obtained at different discharge currents is the maximum discharge capacity of the battery:

$$ {\text{C}}_{ \text{max} } = I_{ 1} \cdot {\text{t}}_{ 1} + I_{ 2} \cdot {\text{t}}_{ 2} + \cdots + I_{\text{n}} \cdot {\text{t}}_{\text{n}} . $$

The equilibrium potentials of LiCoO_2_ electrodes were measured with the pulsed cycle method (3 min with applied current, followed by 3 min rest) in the range of potentials between 2.6 and 4.2 V vs. Li^+^/Li. The equilibrium charge/discharge current was C/10 (15 mA/g). These procedures were described in detail by Spodaryk et al. [53].

The exchange current densities were calculated from the Tafel plot, i.e. dependence of current vs. overpotential. Currents (±i), starting from the smallest to the highest, were alternatively applied and the potentials during the current flow were measured. From the overpotential (the difference between measured potential with the applied current and equilibrium potential, i.e. the potential which the electrode reaches during rest time), the exchange current densities were calculated. The detailed method is described by Chartouni et al. [54].

Electrochemical impedance spectroscopy (EIS) data were obtained using a potentiostat/galvanostat PGSTAT302N with FRA module (Metrohm Autolab). Impedance spectra of the Li-ion batteries were measured in the range of working frequencies from 10 mHz to 100 kHz. The range was built using a logarithmic distribution. The voltage modulation amplitude was 10 mV. The EIS spectra were analysed using fitting procedure in NOVA 1.4 software from Metrohm Autolab. The accuracy of the potentials measurements is ±2 mV, of the current ±2% and of the capacity ±2%.

The values of the elements from the equivalent circuit model (Additional file 1: Figure S10) were obtained by the following formulas:

$$ {\text{Z}}_{\text{Ri}} = {\text{R}}_{\text{i}} $$

where R_i_ is contact resistance or charge transfer resistance, Ohm,Constant phase element (CPE), which models the behavior of an imperfect capacitor or of a double layer, calculated by:

$$ Z_{Q} = \frac{1}{{Y{_0}(j\omega )^{n} }} $$

where Y_0_ is admittance of an ideal capacitance, siemens S; *n* is an empirical constant, 0 < *n* < 1 (*n* is frequency independent and in the case *n* = 1 formula describes an ideal capacitor, *n* = 0—resistor, *n* = 0.5—Warburg impedance); *j* is imaginary part of impedance; ω is angular frequency, rad/s, $\omega = 2\pi f;f$ is frequency, Hz.

The Warburg impedance is provided by:

$$ Z_{W} = \frac{1}{{Y{_0}\sqrt {j\omega } }} $$

#### Lung cell cultures

All in vitro exposure experiments in this study were conducted with a 3D triple cell co-culture model of the human epithelial tissue barrier cultured at the ALI. This system has previously been described in detail [39, 55]. Briefly, the model consists of a layer of human alveolar type II-like epithelial cells (A549, derived from the American Type Culture Collection) with human monocyte-derived macrophages (MDM) on the apical side (upper chamber) and monocyte-derived dendritic cells (MDDC) on the basolateral side (lower chamber). A549 epithelial cells were cultured at a density of 0.5 × 10^6^ cells/ml in cell culture medium RPMI 1640 (supplemented), on BD Falcon cell culture inserts (high pore density PET membranes, 4.2 cm^2^ growth area, 3.0 µm pore size; *Beckton Dickinson AG, Switzerland*). The cell culture densities of MDM and MDDC were 5 × 10^4^ and 25 × 10^4^ cells/insert, respectively [56].

Human blood monocytes were isolated from different, individual buffy coats received from the Swiss blood donation service (Bern, Switzerland) (i.e. different donor for each exposure), using CD14^+^ MicroBeads as described previously [57]. Due to this, variations in the background between different sets of cell cultures were expected to occur. Co-cultures were incubated for 24 h under suspension conditions in order to allow cell–cell habituation. Subsequently, cell culture medium was extracted from the apical layer to allow formation of the ALI over a period of 24 h in the incubator prior to particle exposures.

#### Air–liquid interface cell exposure system

The dry powder insufflator (DP-4, Penn Century, USA) was used to pulverise the LiCoO_2_ particles. The particle exposure system consisted of a closed chamber (15 × 15 × 35 cm) coated with aluminium foil and equipped with a quartz crystal microbalance (QCM) for the in situ determination of the amount of material deposited. As the material settles onto the QCM, the frequency of the crystal changes (ΔF). The ΔF value (Hz) calculated from the recorded frequency values before and after deposition of material is converted to deposited mass per area (μg/cm^2^) as described in [58].

To avoid electrostatic blocking of the needle, aggregation, asymmetric deposition and low deposition yield, a stainless steel needle without bevel of 2 mm Ø and 7 cm of length was used as pulverization means with a gas expulsion flow of ~120 ml/s of air in two pulse of ~0.5 s for each exposure.

#### Particles exposures

**Fig. 1:**
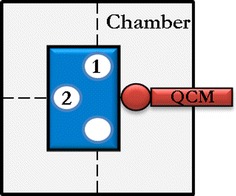
Scheme of the exposure chamber bottom viewed from the top

As described for the aerosolisation of dry volcanic ash particles [59] the pulverisation of the dry powder of nanoparticles produces a radial distribution of the particles at the bottom of the chamber. In order to obtain a regular and reproducible distribution of particles on the cells, the 6-well culture plates were placed in such a way that the inserts holding the triple cell co-cultures and the QCM balance were disposed equidistant from the centre in a cross-like pattern as drawn in the scheme below (Fig. 1).

Two inserts/wells were used for each of the three different concentrations of nanoparticles and microparticles. Experiments were repeated 3–4 times for each of the two particle sizes chosen (micronsize commercial particles and homemade nanoparticles). The pulverisation process took place over a period of about 1 month with each week a different blood donor source.

The samples (wells) were incubated overnight at 37 °C and 5% CO_2_. The day after incubation, the supernatant was removed and replaced with 2 ml of culture medium.

### Biological assays

#### Cytokine and chemokine quantification

The pro-inflammatory response of the triple co-culture after exposure to LiCoO_2_ particles was quantified using the amount of the pro-inflammatory mediators which are tumor necrosis factor α (TNF-α) and interleukin-8 (IL-8) using commercial ELISA development kit and the related supplier protocol. The positive control for the pro-inflammatory proteins was treated with lipopolysaccharide 1 µg/ml (LPS) for 24 h.

#### Optical microscopy/LSM microscopy

After the exposure, cells were fixed and labelled as previously described by Lehmann et al. [56]. In short, samples were stained with a 250 µl mix of a 1:50 dilution of phalloidin-rhodamine for cell cytoskeleton and 1:100 dilution of 4′,6-diamidino-2-phenylindole (DAPI) for the cell nuclei. Coverslips were then mounted onto microscope slides using Glycergel and imaged by LSM.

## Results

### 1-Solid states structures

**Scheme 1:**
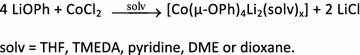
General reaction scheme for obtaining compounds **1**–**7**

Compounds **1**–**7** were obtained by reacting CoCl_2_ with LiOPh in THF, followed by crystallization in THF under different conditions (temperature, presence of water or not, leading to compounds **1**–**3**) or by eliminating the THF solvent and replacing it with other mono- or bis-dentate ligands, like TMEDA, dioxane, DME, or pyridine (**4**–**7**). A general reaction scheme (Scheme 1) resumes the family of compounds obtained. We describe here the single crystal structures of compounds **1**–**5**, on which we base our structural discussion. For compounds **6** and **7**, the single crystal structures could not be determined as the single crystal quality was poor; yet, the chemical analyses confirm a chemical composition in analogy to the other five compounds.

**Fig. 2:**
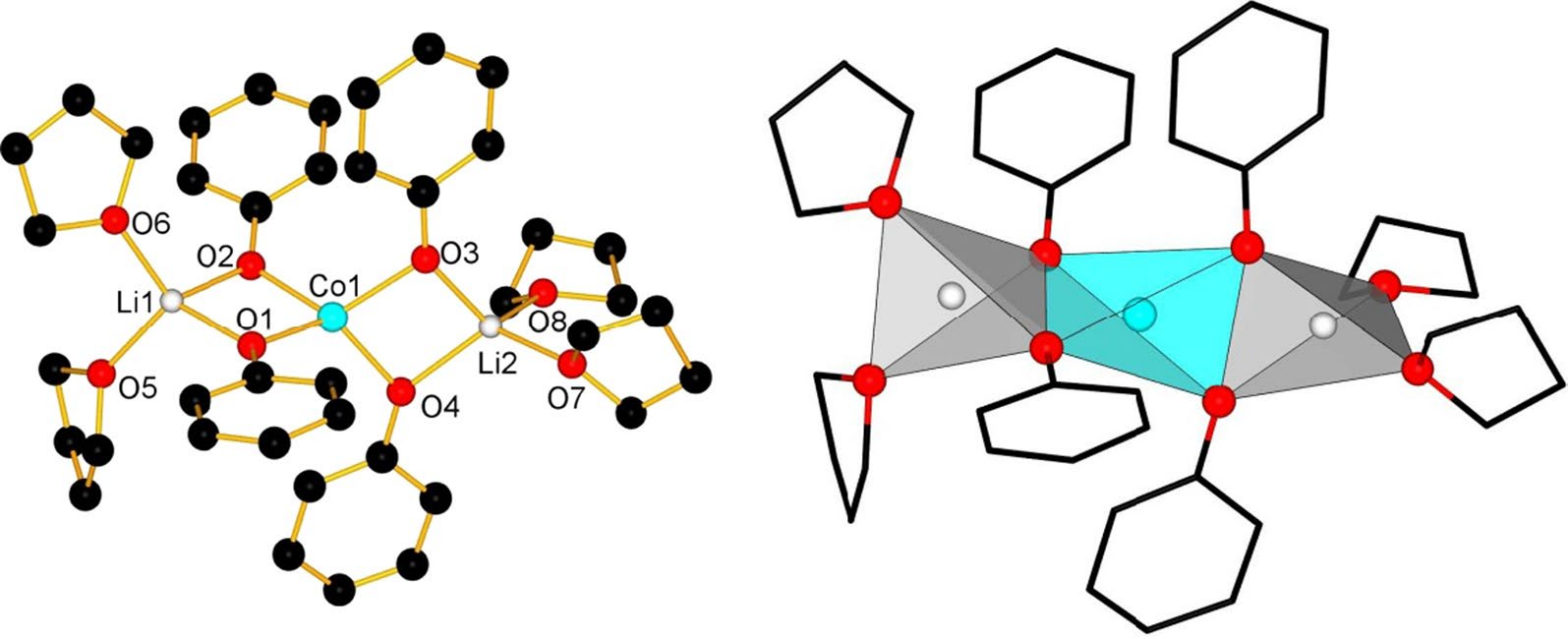
Labelled view of the molecular structure of **1**, H-atoms are omitted for clarity (*left*); coordination polyhedra in **1** (*right*)

Among the compounds, different structure types could be identified depending on the solvent present. For compounds **1**–**7**, the core of the structure is essentially based on one central cobalt ion which is tetrahedrally coordinated by four phenolate entities, bridging pairwise to two lithium ions. The coordination spheres of the lithium cations are completed by coordinating solvent molecules, leading either to molecular entities or a coordination polymer in case of **5**. Figure 2 shows as an example of such a core structure the one of compound **1**. In compound **3**, the terminal ligands of one of the two Li-ions have been formally replaced by two water molecules, which act as bridging ligands between two [Li_2_Co(OPh)_4_] cores, leading thus to a dimer-type structure. Detailed structure descriptions for **1**–**5** with distances and angles are given in the Additional file 1: Text 2, while a resume is given in Table 6.

### Compounds 8–12

**Scheme 2:**
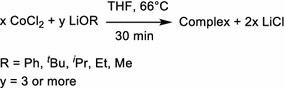
General reaction equation for the synthesis of compound **8**–**12**

For the compounds **8**–**12**, the aim was to test ligands other than aryloxides, such as alkoxides, and to also mix aryloxides and alkoxides as ligands. The synthesis used is similar to the one for compound **1** (Scheme 2), but replacing the LiOPh with alkoxides or using a mix of both.

**Fig. 3:**
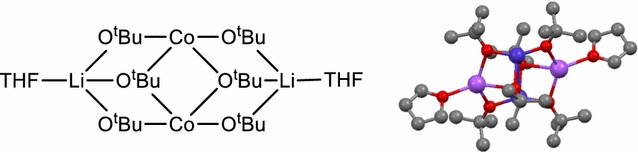
Proposed structure for **8** (*left*) based on the [Na_2_Co_2_(O^*t*^Bu)_6_(thf)_2_] compound (*right*, *dark blue* Co, *violet* Na, *red* O,* grey* C; H-atoms omitted described in [60]

Since the precursor compounds **8, 10, 11** and **12** did not afford single crystals, other methods were used to approach their structure. In possible analogy to compound **8**, the sodium compound [Na_2_Co_2_(O^*t*^Bu)_6_(thf)_2_] was described in the literature [60]. Since the sodium ions are coordinated by four ligands, similar to the preferred coordination of Li^+^, and since Co^2+^ tends to a tetrahedral coordination [61], we propose a similar structure for the lithium compound **8** (Fig. 3). The TGA and NMR measurements confirm that there are two THF molecules per three O^*t*^Bu ligands and the ICP measurement gives a ratio of one lithium for one cobalt ion.

The compounds **10**–**12** were also analyzed by TGA and NMR to determine the amount of ligand and solvent remaining in the solid state structure and the ratio between the ligand and the coordinating solvent molecules. ICP measurements and argentometric titrations of chloride (Additional file 1: Table S3) were also performed to evaluate the ratio of lithium per cobalt ions and the amount of LiCl remaining in the material. The results are resumed in Table 2.

**Table 2:** Combined results from TGA, NMR, ICP and argentometric titration for compounds **8–12**

Compound no—reagent	Ligand eq. vs. Co eq.	Solvent molecules per complex	Free lithium (eq.)	LiCl (eq.)
**8**—LiO^*t*^Bu	3	4 (residual THF)	1 Li per Co	2 Li per Co
**9**—LiO^*t*^Bu + LiOPh	1 + 2 (3)	4	1 Li per Co	2 Li per Co
**10**—LiO^*i*^Pr	3	2 THF	1 Li per Co	2 Li per Co
**11**—LiOEt	6	4–5 THF	4 Li per Co	2 Li per Co
**12**—LiOMe	3	2 THF/2 MeOH	1 Li per Co	2 Li per Co

**Fig. 4:**
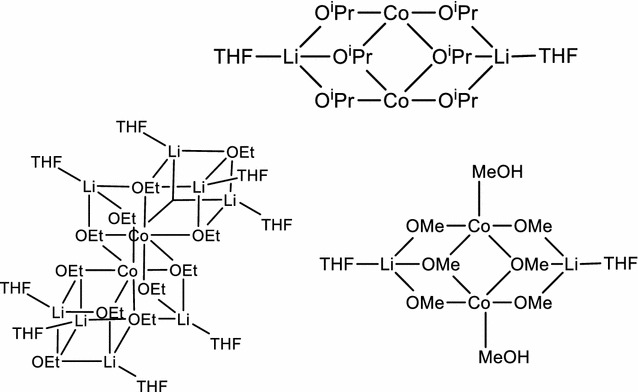
Proposed structure of compound **10** (*top*), **11** (*left bottom*) and **12** (*right bottom*)

From the synthesis, we observed that three equivalents of ligand are required to form carbonate-free LiCoO_2_ from this precursor **10**. The low amount of impurity of mainly Li_2_CO_3_ after combustion indicates that there is no excess of unreacted lithium precursor. We also found one Li^+^ for one Co^2+^ ion in the complex as well as two THF molecules. From this data we propose that the O^*i*^Pr-compound possesses a structure similar to the O^*t*^Bu-precursor **8** (Fig. 4). Using the same method for the compound **12** and based on the findings shown in Table 2, we can propose a similar structure as for **8** (Fig. 4). The extra methanol molecules are difficult to assess since both methanol and THF have almost the same boiling point. Finally, NMR measurements are not helpful since the broadening of the signals (due to the paramagnetic influence of the cobalt ion) hides most of the possible peak shifts.

The compound **11** is the only one which does not follow this rule of three ligands per Co^2+^ and requires six ligands per Co^2+^ to form the desired oxide without impurities of Co_3_O_4_. An open double heterocubane structure is proposed, as it combines the minimum amount of ligands, the amount of free lithium for coordination, the amount of THF and the preferred coordination of lithium ions (4) and cobalt ions (4,6) as determined by TGA, NMR, ICP and argentometric titration (Fig. 4).

**Fig. 5:**
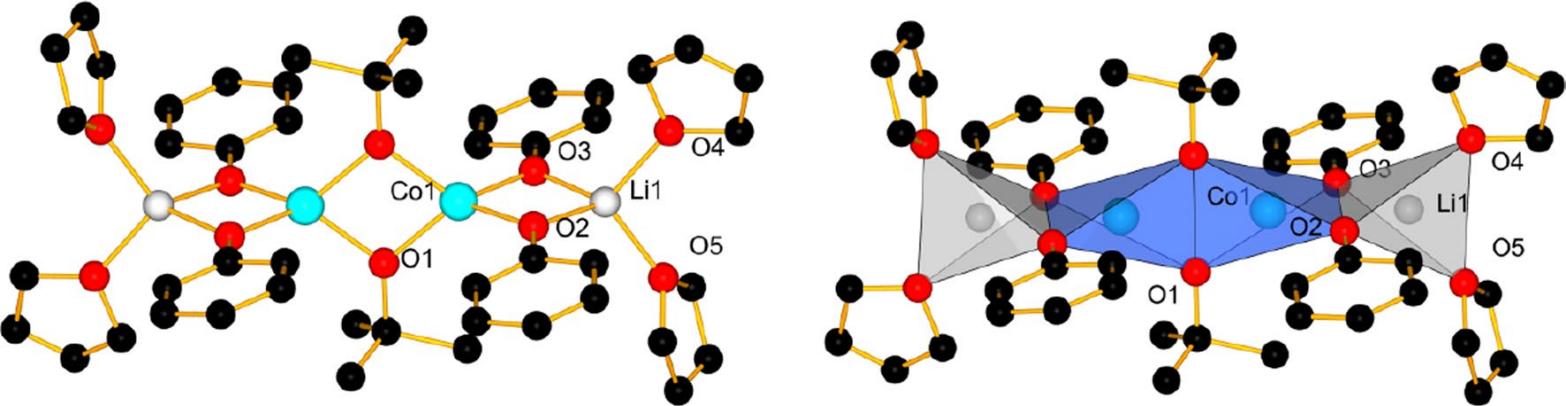
Molecular view of compound **9** measured by XRD. H-atoms are omitted for clarity

Compound **9** is an interesting mixed ligand compound as it forms molecules of [(thf)_2_Li(*μ*-OPh)_2_Co(*μ*-O^*t*^Bu)]_2_ where the two O^*t*^Bu groups act as bridging ligands between two Co^2+^ ions. The OPh ligands bridge pairwise between the cobalt and lithium ions, while two THF molecules complete the coordination of the lithium ions (Fig. 5). A detailed description with distances and angles is given in the Additional file 1: Table S1 and Text 2. The bond valence sums are >2 for both cobalt ions and >1 for both lithium ions, indicating sufficient good coordination of the metal ions by their ligands, as it is also the case for compounds **1**–**5** (Table 6).

### Thermal decomposition to LiCoO_2_

Among all compounds, **2** and **3** are difficult to handle as they lose their solvent molecules very quickly. The compounds **4**–**7** are not well suited for the formation of oxide at low temperature because of their relatively high boiling point, high carbon content and molecular weight. The following investigations for the formation of LiCoO_2_ were thus limited to compounds containing THF and the less carbon containing compounds, hence **1** and **8** to **12**.

**Fig. 6:**
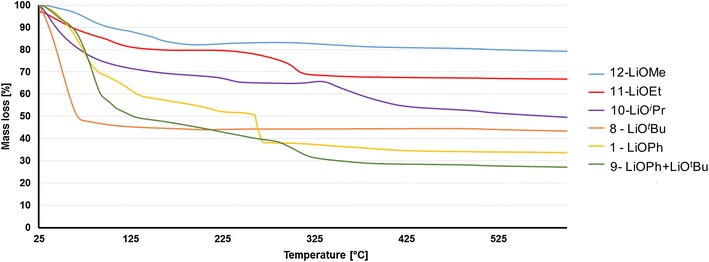
TGA measurements of complexes **1, 8, 9, 10, 11, 12**

In order to use these compounds as precursors for the manufacturing of LiCoO_2_, TGA measurements under oxygen atmosphere with open crucible were performed on the chosen compounds (Fig. 6). The general decomposition process of these complexes begins with the loss of the coordinated and residual non-coordinated solvent molecules before 120 °C (THF B.P. 66 °C, MeOH 65 °C). At higher temperature, between ca. 100 and 400 °C depending on the precursor, the combustion process occurs: it consists of an oxidation of the Co^2+^ to Co^3+^ and of the ligand carbon backbone combustion. Above the temperature of 450 °C, the masses remain quasi constant (Fig. 6). The completed combustion temperature and the detail thermal measurement information are described in Additional file 1: Tables S4 and S5.

**Fig. 7:**
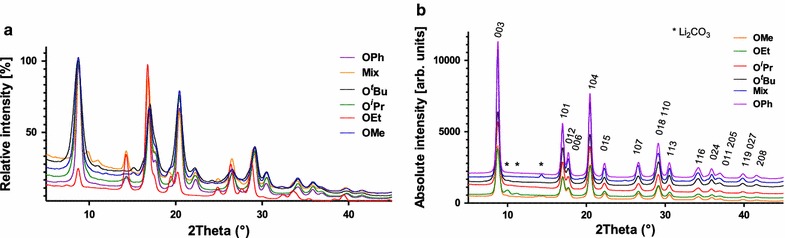
XRD patterns of the oxides obtained after combustion of the precursors **1, 8, 9, 10, 11,** and **12** at 300 °C (**a**) and 450 °C (**b**) in air

Based on the minimum temperature of decomposition of the complexes determined by TGA, combustion tests were performed at different temperatures. Heating to the minimal temperature of decomposition of the precursors of 300 °C for 1 h lead to the formation of the HT-LCO phase with some byproducts (Li_2_CO_3_) (Fig. 7a). Since Li_2_CO_3_ is highly soluble in water, it was removed after rinsing. We believe that the formation of HT-LCO at such a low temperature is possible due to the preorganization of metal ions within the heterobimetallic single source precursors. We decided nevertheless to increase the decomposition temperature by 50–100 °C compared to the decomposition temperature of the compounds in order to reduce the amount of byproducts, and for comparison purposes, the temperature was set to 450 °C for 1 h for all compounds.

After indexation of the powder diffractograms obtained after combustion at 450 °C, all of the tested precursors (**1**, **8**–**12**) afforded LiCoO_2_ with low amounts of impurities that could not be detected by powder X-ray analysis after washing with water, hence less than 5% (Fig. 7). Heating to the minimal temperature of decomposition of the precursors of 300 °C for 1 h leads to the formation of the HT-LCO phase with some byproducts (among which Li_2_CO_3_). A Rietveld refinement of the different diffractograms, taken on a Mo source, was performed to determine the exact phase of the oxide. The lattice cell parameters from the different precursors correspond to a slightly distorted HT-LCO, with the space group *R*-3 m. This small distortion of the unit cells arises from the fact that this material is composed of nanocrystallites which possess a more strain than standard micrometric crystallites. The *c/a* ratio gives also an indication on the general cation ordering of the oxide phase. If the *c/a* ratio is 4.899 or lower, it means that it is a cation-disordered rock salt structure, also called the LT-LCO with a spinel structure (*F*d3 m). Since this ratio *c/a* is higher than this value in all cases, it indicates that the high temperature phase has been obtained for all precursors (Table 3).

**Table 3:** Cell parameters of the LiCoO_2_ formed using different precursors and HT-LiCoO_2_ Ref. [61]

Compound	*a*	*c*	*c/a*	Volume (Å^3^)
HT-LiCoO_2_ [61]	2.8156(6)	14.0542(6)	4.99	96.49(4)
**1** (LiOPh)	2.8193(2)	13.930(3)	4.94	95.88 (3)
**8** (LiO^*t*^Bu)	2.8179(3)	13.949(3)	4.95	95.93(4)
**9** (LiOPh + LiO^*t*^Bu)	2.8139(3)	13.970(4)	4.96	95.79(4)
**10** (LiO^*i*^Pr)	2.8199(1)	13.936(2)	4.94	95.97(2)
**11** (LiOEt)	2.8144(2)	13.942(2)	4.95	95.64(2)
**12** (LiOMe)	2.8199(2)	13.956(3)	4.95	96.11(3)

**Fig. 8:**
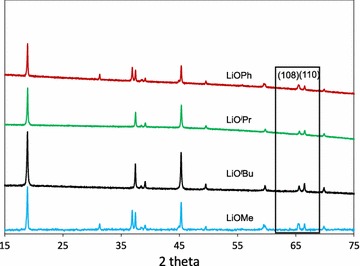
XRD of LiCoO_2_ prepared with **8**-LiO^*t*^Bu, **10**-LiO^*i*^Pr, **12**-LiOMe and **1**-LiOPh precursors

Another method to identify LT and HT phases of LiCoO_2_ is to verify the peaks at 2 theta = 65–67° (λ = Cu-K_α1_). The HT-LCO has two split peaks of the (108) and (110) planes while the LT-LCO has one single peak of the (440) plane at 65° [13, 62]. As shown in Fig. 8 below, all the materials prepared with O^*t*^Bu, O^*i*^Pr, OMe and OPh show two split peaks corresponding to the HT-LCO phase.

**Fig. 9:**
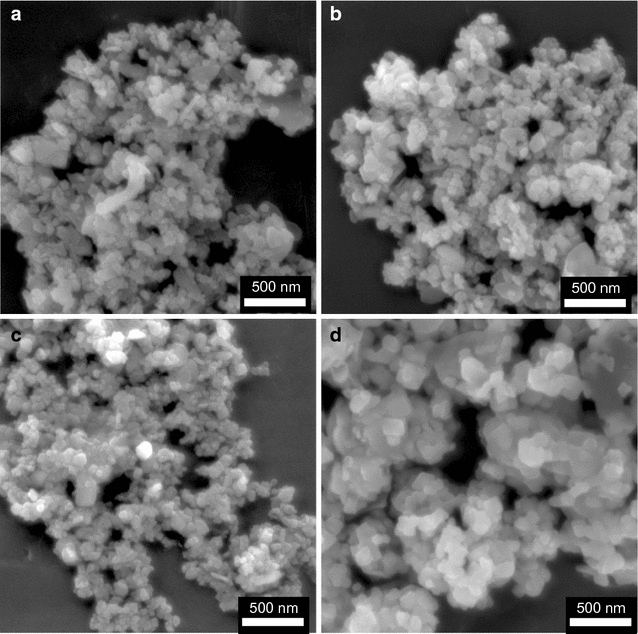
SEM images of LiCoO_2_ prepared with **8**-LiO^*t*^Bu (**a**), **10**-LiO^*i*^Pr (**b**), **12**-LiOMe (**c**), and **1**-LiOPh (**d**) at 450 °C for 1 h

After thermal treatment at 450 °C, the morphologies of the materials prepared with different precursors were analyzed using SEM (Fig. 9). All the materials show polyhedral shapes but the materials obtained from LiO^*i*^Pr and LiOPh precursors formed rhombohedral and triangle shapes.

Since the detection limit in powder X-ray diffraction is 3–5%, Raman spectroscopy was used to complete the analysis. The HT-LCO possesses only two Raman active modes: A_1g_ (Co–O stretching) ʋ_1_ at 595 cm^−1^ and E_g_ (O–Co–O bending) ʋ_2_ at 485 cm^−1^, while LT-LCO has four Raman active modes (A_1g_, E_g_, 2 F_2g_) which are respectively at ʋ = 590, 484, 605 and 449 cm^−1^ and are due to the mixing of cations in the structure [63].

**Fig. 10:**
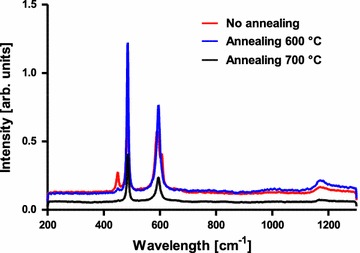
Raman spectra of the annealed nano-LiCoO_2_ obtained from compound **8** at different temperatures and annealing steps (0x = 500 °C for 2 h, 1x = first annealing at 600 °C for 1 h and 2x = second annealing at 700 °C for 30 min)

The Raman spectrum of our non-annealed nano-LCO obtained from compound **8** shows a contamination of the HT-LCO with the LT phase which can be easily removed by annealing at 600 °C for 1 h. No significant improvement can be observed for a 700 °C annealing (Fig. 10). In order to avoid particle growth due to coalescence and ripening, the duration and temperature of annealing have to be minimized, hence we used the 600 °C annealed nanoparticles for the biological assays described later.

ICP measurements on the nano-LCO obtained from **8** and on commercial micron-sized LCO were carried out and the ratio between Li^+^ and Co^3+^ ions was calculated: we found 0.96 ± 0.02 Li^+^ ions per Co^3+^ ion for the nano-LCO (Additional file 1: Table S4). Thus the stoichiometry is a little bit lower than the optimal 1:1 stoichiometry ratio. This can be explained at least partly by the washing steps during which part of the Li^+^ can be washed away, the mechanical stress induced by ultrasounds and the shear stress of the centrifuge and the annealing in which the Li^+^ and Co^3+^ ions can diffuse out of the oxide into the crucible. The ICP measurements of the micro-LCO give a Li^+^ content of 1.01 ± 0.02 which is the optimal ratio for the HT-LCO.

### Morphologies and determination of the particle and crystallite sizes

The crystallite and particle sizes were assessed via the Scherrer equation (X-ray) and the BET equation (gas adsorption), respectively. The details are described in the Additional file 1: Equation S1 – S5.

Table 4 gives the summary of specific surface area, different sizes of particles and crystallites obtained under identical combustion conditions (temperature, time, speed of heating/cooling and atmosphere composition) depending on the starting complexes.

**Table 4:** The specific surface area, mean particle size and crystallite size of LiCoO_2_ prepared with different precursors

SSA (m^2^/g)												
Annealed	**1**-LiOPh		**8**-LiO^*t*^Bu		**9**-(LiOPh + LiO^*t*^Bu)		**10**-LiO^*i*^Pr		**12**-LiOMe		**11**-LiOEt	
500 °C	9.46 (025)		16.50 (0.2)		9.62 (0.2)		11.50 (0.3)		19.70 (0.12)		not measured	
600 °C	2.59 (0.015)		12.50 (0.14)		0.95 (0.03)		3.65 (0.05)		8.00 (0.07)		0.95 (0.01)	
700 °C	0.50 (0.02)		6.10 (0.17)		0.78 (0.02)		3.04 (0.05)		5.50 (0.05)		0.34 (0.02)	

Particle size (P)* and crystal size(C)** (nm)												
	P(**1**)	C(**1**)	P(**8**)	C(**8**)	P(**9**)	C(**9**)	P(**10**)	C(**10**)	P(**12**)	C(**12**)	P(**11**)	C(**11**)
500 °C	126 (2)	50 (2)	72 (1)	60 (2)	124 (2)	75 (2)	103 (2)	40 (1)	60 (1)	50 (4)	Not measured	Not measured
600 °C	459 (2)	45 (2)	95 (1), 40***	45 (3)	1251 (26)	150 (3)	326 (3)	75 (1)	149 (1), 15***	45 (1)	1251 (9)	110 (3)
700 °C	2376 (60)	90 (2)	195 (4)	55 (2)	1529 (26)	185 (1)	391 (4)	295 (1)	216 (1)	170 (2)	3494 (130)	285 (1)

* The mean particle size was determined by the equation of d = K/(ρ×S_BET_), where K is the shape factor, ρ is the density of the material (5.05 g/cc). and S_BET_ is the specific surface area of the material

** Crystal size was determined using Scherrer equation d = Kλ/(B cosθ), where d is the mean crystallite size in volume-weight, λ is the wavelength of the X-rays, B is the width of a peak at a half maximum due to size effects assuming that there is no strain, K is a constant value of 0.89, and θ is the incident angle

*** Particle sizes were obtained after 1 h of ball milling

**Fig. 11:**
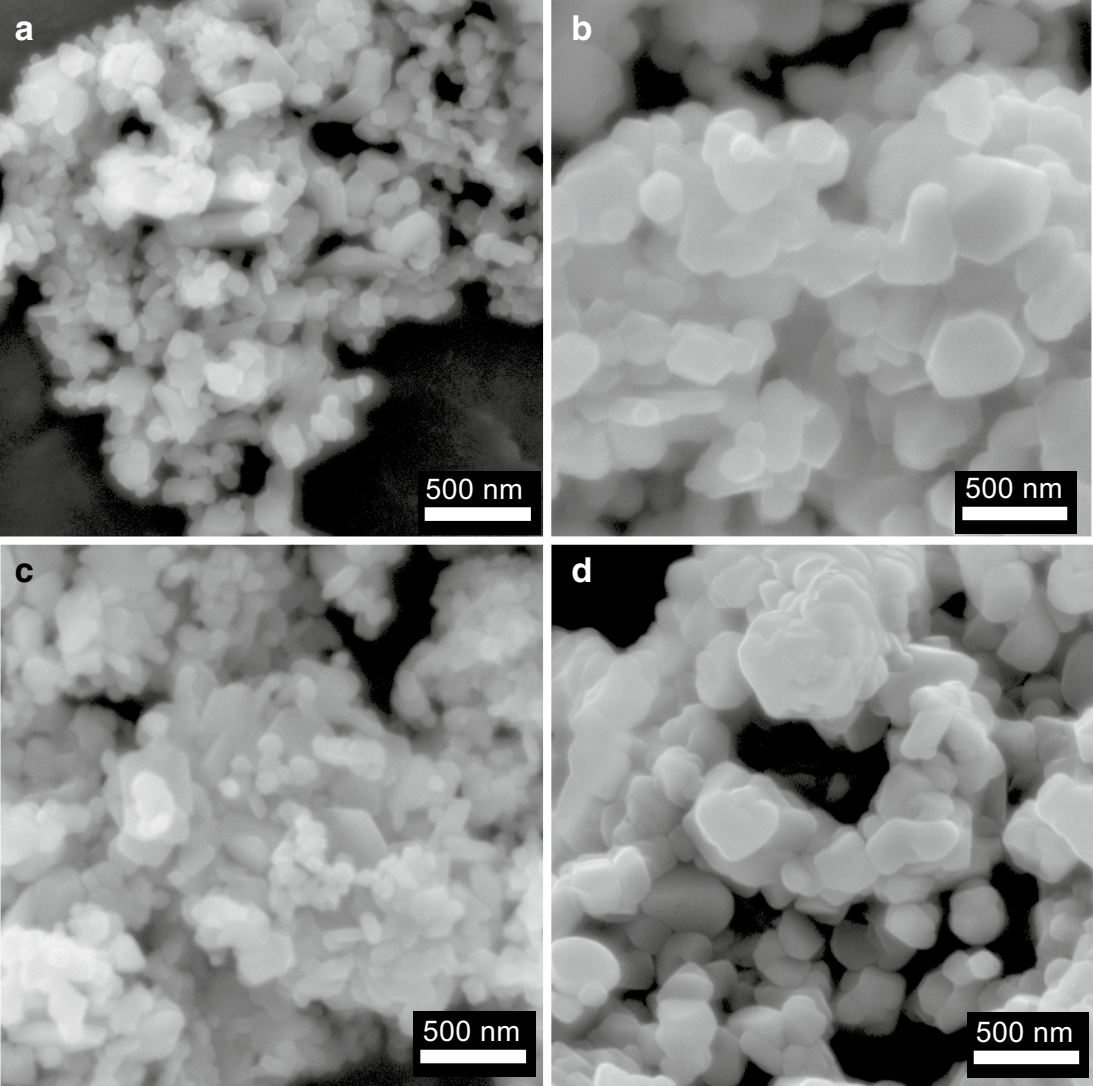
SEM images of LiCoO_2_ materials prepared with LiO^*t*^Bu (**a**), LiO^*i*^Pr (**b**), LiOMe (**c**), LiOPh (**d**) annealed at 600 °C

The morphologies of the particles were investigated by SEM images (Fig. 11). The shapes of the particles obtained from the different precursors are similar and submicron. It is also noted that the material always tends to form large aggregates due to its high surface area.

### Electrochemistry and Li-ion diffusion

Finally, in order to learn if the size of particles has a direct influence on the Li-ion diffusion, cyclic voltammetry of LiCoO_2_ electrodes was performed on two different particles sizes: 40 and 15 nm coming from the precursors **8** and **12**, respectively after a prolonged ball milling of 1 h instead of 15 min.

**Fig. 12:**
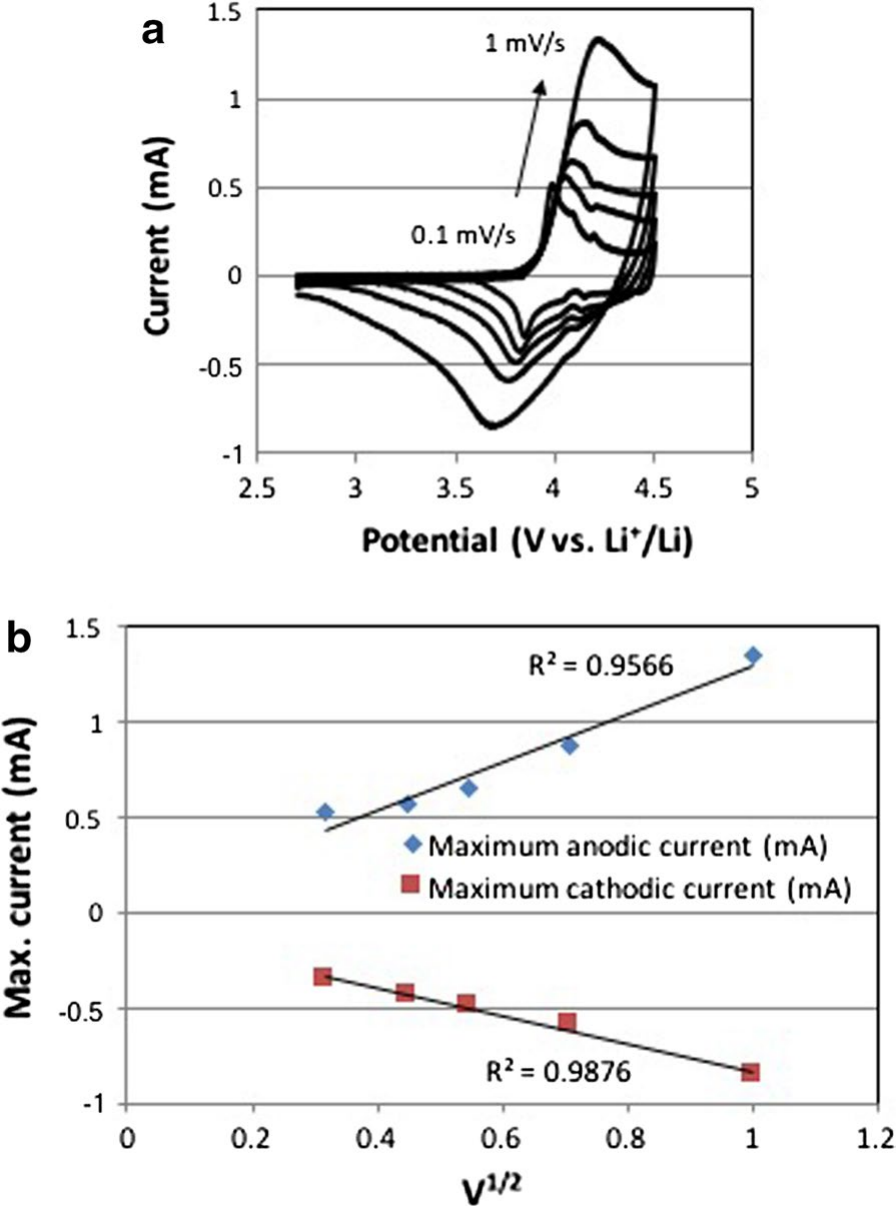
**a** Cyclic voltammograms of the 40 nm LiCoO_2_ particles from compound **8** at different scan rates. **b** The maximum anodic and cathodic current peaks of LiCoO_2_ electrode vs. the square root of sweep rate

Figure 12a shows the cyclic voltammograms of LiCoO_2_ electrode prepared with LiO^*t*^Bu precursor at different scan rates between 0.1 and 1 mV/s. When Li^+^ is extracted from LiCoO_2_, Co^3+^ in LiCoO_2_ is oxidized and electron is released (LiCo^3+^O_2_→Li_1−x_Co^4+/3+^O_2_ + xe^−^ + xLi^+^). On the other hand, oxidized Li_1−x_CoO_2_ is reduced and electron is uptaken when Li^+^ is re-inserted into Li_1−x_CoO_2_ (Li_1−x_Co^4+/3+^ O_2_ + xe^−^ + xLi^+^→LiCo^3+^O_2_). Therefore, the current increased where the redox reactions of Co^3+^/Co^4+^ occurred above 3.9 V for anodic peaks and between 3.6 and 3.9 V vs. Li^+^/Li for cathodic peaks. The CVs and the maximum current peaks of the compound **12** are shown in Additional file 1: Figure S9.

The Li-ion diffusion coefficient can be determined from these cyclic voltrammograms by using the Randle–Sevcik equation. The Randles–Sevcik equation [63]:

1 $$ {\text{Ip}} = \left( { 2. 6 9 { } \times { 1}0^{ 5} } \right){\text{ n}}^{ 3/ 2} {\text{A D}}_{\text{Li}}^{ 1/ 2} {\text{C v}}^{ 1/ 2} $$

with Ip the peak current; n the number of transfer electrons; A the surface area of the electrode; C the concentration of reactants; and v the scan rate.

The plot of the square root of the scan rate vs. the anodic or cathodic peaks gives the slopes which represent the square root of the Li^+^ ion diffusion coefficient value, D_Li+_ (Fig. 12b).

The Li^+^ ion diffusion coefficients (D_Li+_) of our nanoparticles were 2.3 × 10^−5^ and 4.5 × 10^−6^ cm^2^ s^−1^ for **8**-LiO^*t*^Bu and **12**-LiOMe, respectively while the one of commercial HT-LCO was 2 × 10^−7^ cm^2^ s^−1^ (Table 5). The values obtained from nanoparticles are 20–100 higher than the standard value for HT-LCO [64]. Thus the kinetics with Li^+^ ions are much faster in nanoscale LCO than in micron-LCO. When we compare the values of diffusion coefficients of 15 and 40 nm of nano-LCO, the larger particle size of 40 nm has even higher diffusion coefficient. It will be explained in the discussion part later.

**Table 5:** Size and Li-ion diffusion coefficient comparison between two precursors, **8** and **12**, and HT-LCO Ref. [64]

Compounds/precursors	Size	D_Li_ (cm^2^ s^−1^)
HT-LCO [64]	11 μm	2 × 10^−7^
**8**—LiO^*t*^Bu	40 nm	2.3 × 10^−5^
**12**—LiOMe	15 nm	4.5 × 10^−6^

### Electrochemical properties

**Fig. 13:**
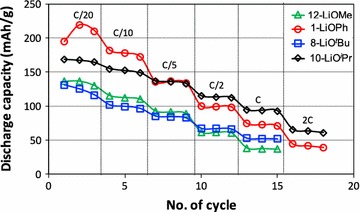
Discharge capacities of LiCoO_2_ electrode. LiCoO_2_ materials were prepared by **8**-LiO^*t*^Bu, **10**-LiO^*i*^Pr, **12**-LiOMe and **1**-LiOPh

After D_Li+_ was determined, the battery properties of our nanoscale LCO materials were investigated. The charge/discharge current is expressed as a C-rate to evaluate battery capacities at various current values. A C-rate is a measure of the rate at which a battery is discharged relative to its maximum capacity. The current density and C-rate are determined by the nominal specific capacity of 150 mAh/g. For example, the current densities are 150 and 7.5 mA/g at 1C (a battery is charged in 1 h) and C/20 (a battery is charged in 20 h), respectively. Figure 13 shows the discharge capacities of LiCoO_2_ electrodes prepared by the precursors **1**-LiOPh, **8**-LiO^*t*^Bu, **10**-LiO^*i*^Pr and **12**-LiOMe. Depending on the precursor used in the synthesis, the specific capacity varies. **10**-LiO^*i*^Pr and **1**-LiOPh derived LiCoO_2_ electrodes obtained superior capacities to the ones obtained with **8**-LiO^*t*^Bu precursors. The mean specific capacity of LiCoO_2_ derived from **1**-LiOPh was 210 mAh/g at C/20, which is 77% of the theoretical capacity of 272 mAh/g, while LiCoO_2_ from the LiO^*t*^Bu precursor had 124 mAh/g (46% of the theoretical value) at the same rate.

**Fig. 14:**
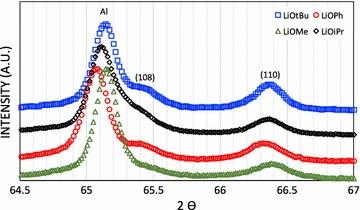
XRD of LiCoO_2_ electrodes after cycling. LiCoO_2_ materials were prepared with different precursors: 8-LiO^*t*^Bu (*square*), 10-LiO^*i*^Pr (⋄), 12-LiOMe (*triangle*) and 1-LiOPh (*circle*) precursors; the aluminum peak stems from the current collector of the electrode

After cycling of charge/discharge at different current densities, we disassembled the batteries for all four samples and rinsed the LiCoO_2_ electrodes to verify their structures. XRD in Fig. 14 shows that all the cycled LiCoO_2_ electrodes have two peaks at (108) and (110) corresponding to the HT-LCO phase, hence the structure is unchanged after cycling.

**Fig. 15:**
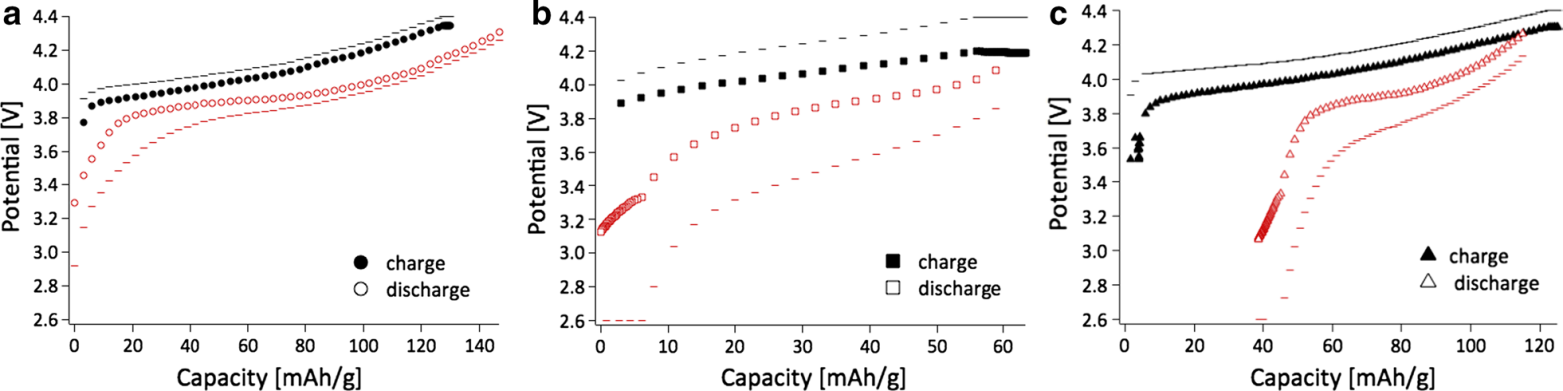
Charge (*filled markers*) and discharge (*empty markers*) curves of LiCoO_2_ electrodes prepared with *filled circle*, *open circle*—LiOPh (**a**), *filled square*, *open square*—LiO^*t*^Bu (**b**), *filled triangle*, *open triangle*—LiOMe (**c**) precursors.* Lines* (-) correspond to the potentials with applied current and markers to the potentials without current (in equilibrium state)

The equilibrium charge/discharge curves of the LiCoO_2_ electrodes obtained from LiOPh, LiO^*t*^Bu and LiOMe precursors were investigated as shown in Fig. 15. The markers are measured when the current is not applied to the battery while the dashed lines are recorded when the current is applied. They show the plateau of equilibrium charge curves at 3.9 V and discharge at 3.8 V vs. Li^+^/Li. The coulombic efficiency of the LiCoO_2_ electrodes from LiOPh reached >95% with relatively low polarization between charge and discharge process (Fig. 15a). In case of the LiCoO_2_ electrode from LiO^*t*^Bu (Fig. 15b), the coulombic efficiency reached also >95% but both charge and discharge processes result in half of the capacities compared to these of the electrodes from LiOPh. Moreover, the potentials during charging with the applied current (dashed lines on the graphs) are higher in Fig. 15b compared to these in Fig. 15a, c.

**Fig. 16:**
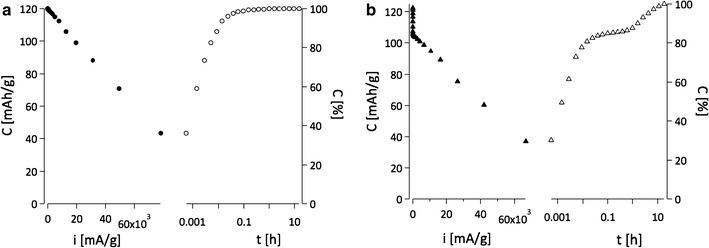
Deep discharge curves for electrodes obtained from: **a** LiOPh (*filled circle*, *open circle*) and **b** LiO^*i*^Pr (*filled triangle*, *open triangle*) *Right axes* indicate the state of discharge in percentage (*empty markers*)

The deep discharge process was evaluated to estimate how fast the battery can reach the maximum discharge capacity of the different LiCoO_2_ electrodes. Figure 16 exhibits that the LiCoO_2_ electrode from LiOPh precursor, (a), can reach 99% of its maximum capacity (120 mAh/g) within 9 min (at 5.2 C) due to the fast kinetic reaction of Li^+^ ion insertion/extraction. Of course, this maximum capacity remained at any lower current densities, showing the plateau on the right side in Fig. 16a. On the other hand, the electrode from LiO^*i*^Pr precursor, (b), can be discharged to 90% of its maximum capacity (122 mAh/g) at much lower current density of 0.44 C (about 26 min) than (a). (b) can reach 85% (104 mAh/g) of its maximum discharge capacity within 6 min (at 7 C). Thus, this deep discharge measurement supports that the discharge capacities at higher current densities (>C/2) are lower in LiCoO_2_ electrode with LiOPh than those in LCO with LiO^*i*^Pr (Fig. 16). Therefore, the kinetics of the electrode (a) obtained from LiO^*i*^Pr is an order of magnitude faster than (b) (obtained from LiOPh) at high current densities.

**Fig. 17:**
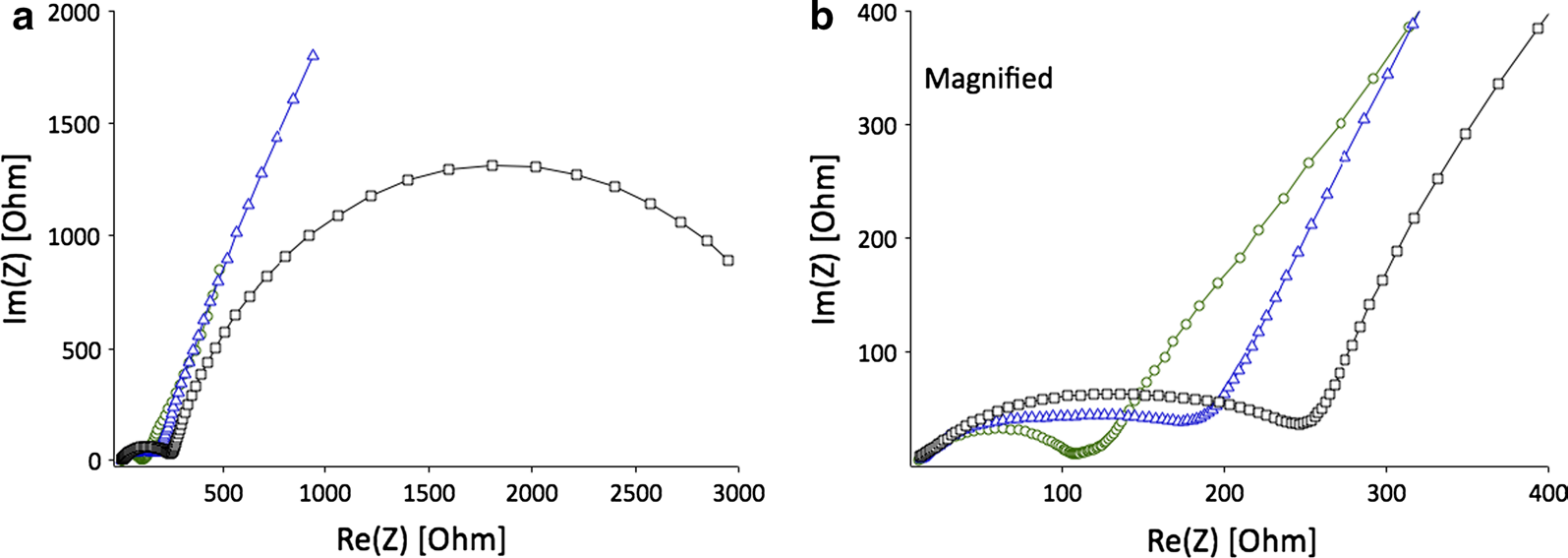
**a** Nyquist plots of coin cells consisting of LiCoO_2_ electrodes with different precursors: *square*—O^*t*^Bu, *triangle*—OMe, *circle*—OPh. **b** Magnified Nyquist plots of (**a**)

The Nyquist plots presented for electrodes with different precursors were obtained in the frequency range of 100 kHz–0.01 Hz at 25 °C (Fig. 17). The EIS spectra of the electrodes with LiOPh and LiOMe precursors are similar in shape with one semicircle and Warburg branch, while the electrode obtained from LiO^*t*^Bu precursor shows hodographs with two semicircles without Warburg impedance. After fitting the EIS data, the equivalent circuit models were proposed (Additional file 1: Figure S11).

The ion transfer resistance and total impedance of electrodes with different precursors increase in the following sequence: LiOPh < LiOMe < LiO^*t*^Bu, which is in good agreement with the discharge capacities and equilibrium charge/discharge curves.

### Hazard assessment of particles

#### Particle aerosolisation

Nanoparticles obtained from precursor **8**, which was annealed at 600 °C for 1 h, were compared to a commercially obtained, micron-sized LiCoO_2_ sample. A dry powder insufflator was used to aerosolise both materials for direct deposition onto the surface of the multicellular epithelial tissue barrier model. Initially, following aerosolisation, the deposition of the two particle types was characterised in terms of their mass deposition, particle size, as well as their distribution and morphology.

**Fig. 18:**
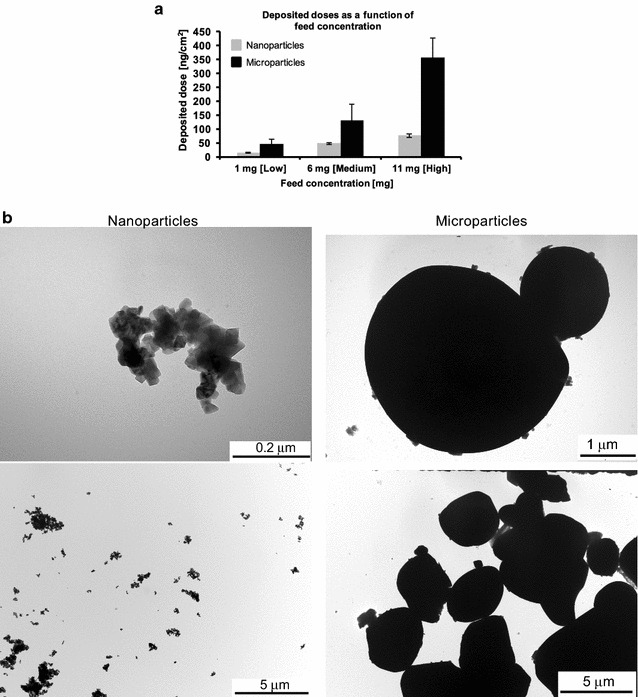
Deposition characterization of aerosolised nano-sized and micron-sized particles. **a** Average mass deposition (ng/cm^2^) of particles quantified using a quartz crystal microbalance (QCM) following nebulisation of low (1 mg), medium (6 mg) and high (11 mg) particle doses using a dry powder insufflator. Data are presented as the mean ± standard error of the mean. **b** Transmission electron micrographs of aerosolized nano- (*left*) and microparticles (*right*), indicating, in a qualitative manner, the heterogeneity of the particle deposition for each particle-size. Images also show a representative overview of the particle morphology following the aerosolisation process

The cell-delivered dose was monitored using an integrated quartz crystal microbalance (QCM) and showed a dose-dependent deposition of the both samples, i.e. 0.81 ± 0.2, 0.55 ± 0.14 and 0.16 ± 0.05 µg for nanoparticles, and 3.92 ± 0.78, 1.46 ± 0.63 and 0.51 ± 0.18 µg for microparticles. It was, however, not possible to achieve the same range of deposited concentrations for both nano- and micron-sized particles despite using the same initial feed concentration, as shown in Fig. 18a. Reason for this, apart from the different pulverisation methods, is that the microparticles can be considered to exhibit a higher density, and therefore greater tendency to agglomerate/aggregate leading to a higher surface density compared to the limited agglomeration/aggregation shown by the nanoparticles.

By using TEM it was observed that the pulverized nanoparticles of LCO formed agglomerates/aggregates ranging from nano-sized to micron-sized (ca. 0.05–50 µm). This could possibly be attributed to the low surface charge of the material (i.e.≤ ±10 mV). The average size of primary nanoparticles was estimated to be 64 ± 5 nm, as determined by the BET method, while the crystallite size was determined to be 60 ± 5 nm using the Scherrer equation. The micron-sized particles were noted to exhibit a size of 10–12 μm, as previously reported [16–21]. In terms of their morphology, nanoparticles were observed to show rhombohedral/tetrahedral shaped patterns, whereas the commercial microparticles were found to be irregular in shape, with most showing roundish shapes (Fig. 18b).

#### Cell death

**Fig. 19:**
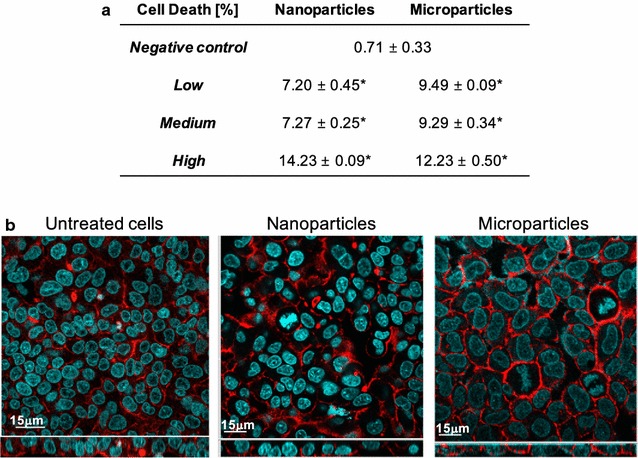
Percentage (%) cell death levels and morphological analysis of the multicellular model of the epithelial tissue barrier following 24 h exposure to both LiCoO_2_ nano-sized and micron-sized particles. **a** *Table* shows quantification of the average % cell death levels of propidium iodide stained cells at the three tested concentrations (low, medium and high), as analysed via one-colour flow cytometry analysis. *Asterisks* indicates a statistically significant increase in the % level of cell death within the multicellular in vitro system compared to the negative control (i.e. cell culture medium only) (*p* > 0.05) (n = 3). **b** Confocal laser scanning microscopy images show F-actin cytoskeleton (*red*) and the nuclei (*blue*) staining of the complete multicellular model following exposure to both particle sizes/types at the highest concentration tested after 24 h

After 24 h exposure, LiCoO_2_ nanoparticles showed limited ability to cause cell death following their aerosolisation onto the in vitro multicellular epithelial tissue barrier model at each particle concentration tested (Fig. 19a). Both low and medium nanoparticle concentrations showed similar effects, whilst the highest concentration applied increased the level of cell death by 50% compared to the lower concentrations studied. This result can be attributed to an ‘overload’ scenario upon the cells at the highest concentration applied (Fig. 19b) [65]. It is important to note that although these values are significantly different from the negative control (*p* > 0.05) (i.e. cell culture media only), with the highest concentration applied showing a maximum of <15% cell death in the in vitro co-culture system, the findings indicate that the nanoparticles are not causing complete destruction of the cellular system but do induce a limited cytotoxic effect at these concentrations. Similar results were also evident following micron-sized LiCoO_2_ particle exposures at each test concentration (Fig. 19a). In respect to these semi-quantitative results, it is also important to highlight that qualitative assessment, via confocal laser scanning microscopy, showed no morphological changes to the multicellular system following exposure to either particle type at the highest concentration applied for 24 h (Fig. 19b).

#### (Pro-)inflammatory response

**Fig. 20:**
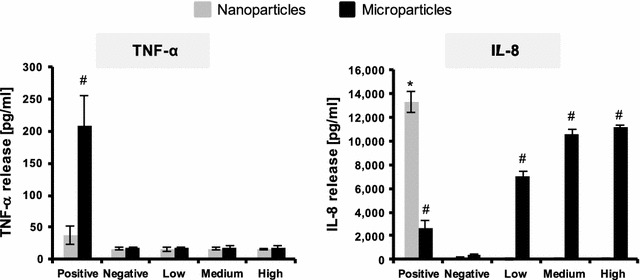
(Pro-)inflammatory response of the multicellular epithelial tissue barrier following 24 h exposure to nano-sized and micron-sized nanoparticles at the three different test concentrations. *Graphs* show the results for the specific (pro-)inflammatory mediators chosen; tumor necrosis factor-α (TNF-α) and interleukin-8 (IL-8). Lipopolysaccharide ([100 µl of 1 µg/ml]) served as the positive assay control, whilst the negative control was cell culture medium only. Data is presented as the mean ± standard error of the mean. ^#^indicates a statistically significant response (*p* > 0.05) compared to the negative control

No significant (pro-)inflammatory response (i.e. either TNF-α and IL-8 release) was observed following nanoparticle exposures across all concentrations tested (Fig. 20). Similar results were observed with the micron-sized particles in terms of the TNF-α response from the multicellular system after 24 h exposure. However, microparticle exposures did show a significant increase (*p* > 0.05*)* in terms of the IL-8 response from the co-culture, in a concentration-dependent manner at this time point (Fig. 20).

## Discussion

**Fig. 21:**
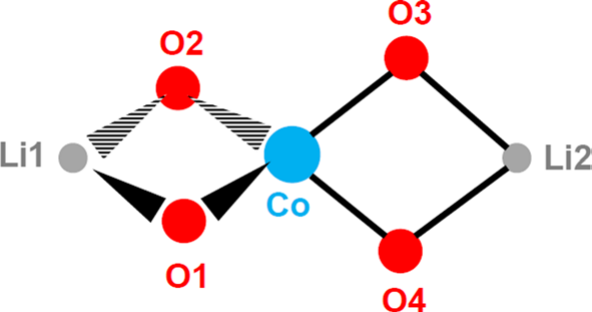
Schematic representation with numbering of the Li_2_Co(OPh)_4_-core of compounds **1**–**7**

The general reaction of CoCl_2_ and LiOPh for the generation of the precursors **1**–**7** is based on the LiCl-elimination and the formation of a mixed phenoxide with always the same metal ion ratio of 2:1 for Li:Co, as found in the core [Li_2_Co(OPh)_4_] of the structures **1**–**7**. The formation of this type of compound is in our hands independent of the amount of LiOPh added (between 1 and 6 equivalents). The core is always made of a central Co^2+^ ion which is surrounded in a (more or less distorted) tetrahedral way by four phenoxide ligands. Two by two, these O-donors act each as μ-bridging ligands to one Li^+^ ion. The coordination sphere of the latter is then completed by either mono- or bidentate donor molecules stemming from the solvent. These coordinated solvent molecules influence the arrangement of the complexes with respect to each other. For instance, 0-dimensional compounds are obtained with monodentate terminal ligands like THF and pyridine or bidentate terminal ligands like DME and TMEDA, whereas bridging ligands such as dioxane lead to polymeric arrangements. In the [Li_2_Co(OPh)_4_] cores (Fig. 21) of all compounds **1**–**5**, for which the single crystal structures could be determined to satisfaction, the Co–O distances are between 1.938(4) and 1.978(4) Å long, while the angles O1–Co–O2 and O3–Co–O4 are very similar with 86°(±1°). The O2–Co–O3 and O1–Co–O4 angles are however more sensitive to the environment of the Li^+^ cations (see Table 6), respectively packing effects, and vary between 112 and 127°.

**Table 6:** Comparison of compounds **1** to **5 and 9**

	**1**	**2**	**3**	**4**		**5**	9
Tetrahedral volume of Co (Å^3^)	3.332	3.384	3.344	3.364		3.339	3.229
Quadratic elongation	1.093	1.088	1.092	1.079		1.100	1.120
Angle variance (°^2^)	378.27	360.86	374.83	322.26		401.40	486.42
O1–Co (Å)	1.961 (6)	1.960 (7)	1.95 (1)	1.954 (3)	1.958 (4)	1.954 (4)	1.947 (3) 1.949 (3)
O2–Co (Å)	1.948 (5)	1.957 (6)	1.93 (1)	1.963 (4)	1.955 (3)	1.962 (4)	1.960 (3)
O3–Co (Å)	1.963 (5)	1.946 (6)	1.961 (8)	1.954 (3)	1.966 (3)	1.978 (4)	1.958 (4)
O4–Co (Å)	1.972 (6)	1.960 (6)	1.966 (7)	1.952 (4)	1.961 (4)	1.938 (4)	/
Mean O–Co (Å)	1.961	1.956	1.952	1.956	1.960	1.958	1.953
O1–Co–O2 (°)	84.9 (2)	86.5 (3)	85.7 (1)	86.8 (2)	86.6 (2)	85.6 (2)	80.2 (1)
O3–Co–O4 (°)	86.0 (2)	85.2 (3)	85.1 (4)	87.5 (1)	87.0 (2)	84.8 (2)	83.9 (2)
O1–Co–O4 (°)	122.4 (2)	121.0 (3)	125.6 (4)	118.6 (1)	122.7 (2)	126.5 (2)	124.4 (2)
O2–Co–O3 (°)	118.4 (2)	120.0 (3)	123.0 (4)	117.6 (2)	125.0 (1)	112.4 (2)	125.2 (1)
Mean O–Co–O (°)	85.45 120.4	85.85 120.5	85.4 124.3	86.98 120.98		85.2 119.45	82.0 124.8
BVS on Co	1.93	1.96	1.98	1.96	1.94	1.95	1.97
BVS on Li	1.17 1.14	1.13 1.14	1.13 1.07	1.19 1.17	1.13 1.14	1.19 1.25	1.18

The difference of composition between **1** and **2** originates from the crystallization technique. Indeed, **1** is prepared at room temperature with the addition of heptane for crystallization, while **2** is crystallized without any co-solvent at −24 °C. These two different methods give two different products: one thermodynamic compound **1** and one kinetic compound **2**, which can be considered as solvates to each other [66].

In the structure of the compounds **1** to **7,** an inherent stoichiometric ratio of two Li^+^ for one Co^2+^ exists, hence excess of one equivalent Li^+^ with respect to the desired LiCoO_2_. During the firing, this excess of Li^+^ in the precursor tends to form lithium carbonate either by reaction with the CO_2_ in air or with the byproducts of the combustion. The carbonate can clearly be seen in powder X-ray diffractogram of the raw oxide. However, these impurities, as well as the main byproduct LiCl (formation of the precursors), can be easily washed away with water. Successful removal of LiCl was confirmed by powder X-ray diffraction as well as TEM/SEM.

For the compounds **8**–**12**, except **11**, the stoichiometric ratio is 1:1 for Li^+^ to Co^2+^, thus there is no excess Li^+^ and hence almost no formation of lithium carbonate (Additional file 1). While we produced our nanoscale materials in quite pure form by this washing step, the analysis of the commercial HT-LiCoO_2_ shows that it contains some Li_2_CO_3_ impurities, which is one of the reactants of its synthesis.

The main physical/chemical differences in the final oxides obtained at 450 °C from **1** and **8**–**12** are the amount of impurities due to stoichiometric reasons and the size of the particles/crystallites obtained. Indeed, the LiOPh precursor **1** tends to form more impurities (carbonates, XRD in Additional file 1: Fig. S7) and a larger crystallite size. The amount of impurity is mainly due to the incorrect stoichiometric ratio in the starting structure of 2:1 for Li:Co, but also to a large amount of carbon atoms in the precursor. However, by decreasing the number of carbon atoms using alkoxide and by balancing the ratio between Co and Li to 1:1, better results in terms of size and smaller amounts of byproducts can be achieved. As shown in the Table 4, sizes as low as 60 nm of HT-LCO can be obtained.

We observed different LCO morphologies from the single source precursors. This could be related to the formation of LCO nuclei, which likely depend on the initial structure of the complex precursor. Not only the core structure, but also the arrangement of the molecules with respect to each other may play a role in the formation of different nuclei.

The redox potentials indeed confirm that the obtained nano-LiCoO_2_ is in the HT-LCO phase. We also recognized that the oxidation of Co^3+^ to Co^4+^ (corresponding to Li^+^ extraction from Li_1−x_CoO_2_) shows higher current than the reduction of Co^4+^ to Co^3+^ (Li^+^ insertion into Li_1−x_CoO_2_). The cyclic voltammograms (CV) of both samples obtained from **12** and **8** show a HT-LCO CV profile with a low polarization and high potential, as expected from the X-ray diffraction pattern.

In terms of the Li^+^ diffusivity, hence the kinetic with respect to Li^+^ ions, we found it to be much faster in nanoscale LCO than in micron-LCO. In other words, the amount of Li^+^ ions available for electrochemistry is larger in nanoscale LCO than that in micron-LCO due to the shorter path length of the Li^+^ ion diffusion. The values obtained are >20 times higher than the standard value for HT-LCO [64]. In the best case measured in our hands, 77%, of all Li^+^ ions were extracted from and re-inserted in the structure of nano-HT-LCO, while for the commercial material, only about 50% of Li^+^ ions (0 < x < 0.5, Li_1−x_CoO_2_) can be used electrochemically in the rhombohedral layered structure of LiCoO_2_. Further de-lithiation of commercial, micro-HT-LCO induces a phase transformation to the monoclinic system [17], resulting in irreversible capacity loss upon cycling. Therefore, the phase stability of LiCoO_2_ is important during lithiation and de-lithiation in order to obtain high coulombic efficiency and longer cycleability of battery. This is what we could show for the nano-HT-LCO after battery cycling by analyzing the material by XRD. Hence, our LCO materials prepared by heterobimetallic single source precursors are stable upon cycling and provide fast electrochemical reactions with Li^+^ ions due to nanosized particles. The LCO materials prepared from various complexes showed different specific capacities. This difference may be related to several parameters such as the homogeneity of particle size, ball milling and the shape of LCO particles. Also, when a particle size distribution is broad, the specific capacity can be less good than the one from the narrower size distributed particles. The large size difference can lead to different Li^+^ diffusion kinetics. However, the larger particles can be broken during ball milling and the size distribution becomes narrower, improving the kinetics of Li^+^ diffusion and finally the specific capacity. The shape of LCO particle can also affect the diffusion of Li^+^ because Li^+^ diffuses in a specifically oriented layer of the structure.

The smaller particle size provides higher diffusion kinetics with Li^+^ because the higher surface area of nano-LiCoO_2_ provides more Li^+^ ions to be released and uptaken into/from the electrolyte. In addition to the high surface area, there is another parameter governing the diffusion kinetics, which is the orientation of Li^+^ diffusion path in the lattice structure of LiCoO_2_. Li^+^ is located in one layer of the LiCoO_2_ lattice cell, diffusing in one preferred orientation. Thus, the length of Li^+^ diffusion path in LiCoO_2_ also affects the diffusion kinetics. We reported that the diffusion of Li^+^ is not only related to the size of particle but also the shape of particle due to the preferred diffusion direction and its length in the lattice structure [67, 68]. In this regard, the higher diffusion coefficient of 40 nm (compound **8**) is probably coming from the shorter diffusion path of Li^+^ in a single particle although the compound **12** has a smaller size of 15 nm.

We also found that LiCoO_2_ produced from LiO^*t*^Bu has a larger overpotential and higher resistance than the one obtained from LiOPh. On the other hand, the LiCoO_2_ electrode formed from LiOMe reached >120 mAh/g of charge capacity. However, the discharge capacity was 90 mAh/g with 70% of coulombic efficiency. These differences of equilibirum charge/discharge curves can be explained by different kinetics at equilibrium state.

The deep discharge measurement supports that the discharge capacities at higher current densities (>C/2) are lower in LiCoO_2_ electrode with LiOPh than those in LCO with LiO^*i*^Pr (Fig. 16). Therefore, the kinetics of the electrode (a) obtained from LiO^*i*^Pr is an order of magnitude faster than (b) (obtained from LiOPh) at high current densities.

The electrochemical properties of batteries are influenced by not only the active material but also the composite, consisting of carbon and the active material [69]. The structural morphology and the physicochemical properties of composite affect the electron transfer and lithium ion diffusion in the electrode [64]. An ongoing follow-up study is hence the optimization of the electrode composites for each nanoscale HT-LCO material as a function of precursor.

In terms of the biological assessment, such studies had never been done on nanoscale LCO and are quite rare for battery materials in general. We found both nano- and micro-LCO to be relatively low toxic in the lung model which we used. The (pro-)inflammatory response upon exposure to nano-LCO was nil across all tested concentrations, while it was dose-dependent for micro-LCO. Neither nanoparticles nor micro-LCO induce a cytotoxic effect at the tested concentrations which leads to more than 15% cell death. In terms of the surface charges of nano and microparticles, we estimate it is low since both particles rather stick together [70].

## Conclusions

A series of 12 new precursors containing lithium and cobalt ions in ratios of 2:1 or 1:1 with different aryl- and alkoxide ligands have been prepared and characterized. Their thermal decomposition leads to the formation of nanoscale HT-LiCoO_2_ with the size of the so obtained nanoparticles depending on the precursor. Also, precursors with a 1:1 ratio of Li^+^ to Co^2+^ lead to quite pure product, while the precursors with a 2:1 ratio gave Li_2_CO_3_ as byproduct. The use of our precursors allowed lowering the production temperature and time for the generation of HT-LiCoO_2_ as a preorganisation of the metal ions takes place in the starting material. The nanomaterials of LiCoO_2_ showed a superior Li-ion diffusivity by a factor of 20–100 compared to commercial LiCoO_2_, depending on the precursor used to generate the cathode material. The electrochemical performance was varied depending on the precursors. LiCoO_2_ with LiOPh and LiO^*i*^Pr provided higher specific capacities while LiCoO_2_ with LiOMe and LiOtBu obtained lower specific capacities. Lithium ion diffusion coefficients of our nanoscale LiCoO_2_ were >10 times higher than the one of microscale LiCoO_2_ due to the shorter path length of lithium ion diffusion in nanomaterial of LiCoO_2_. This means that high surface area of nanoscale LiCoO_2_ can release and take Li^+^ ions much more than micron LiCoO_2_ material at the same condition.

To mimick conditions of recycling of batteries, nanopowders of LiCoO_2_ were tested on a lung cell model. During the spraying of the powders, it was shown that the nanopowders tend to aggregate during the process due to a low zeta-potential. Nevertheless, they are slightly more toxic than the micron-scale material, while toxicity remained overall very low.

## Additional file

**Additional file1: Text 1.** Synthesis of bimetallic compounds.** Table S1.** Crystal data.** Text 2.** Single crystal structure descriptions.** Text 3.** Argentometric titration.** Table S2.** Idealistic oxidation reactions of two types of compounds, precursors** 1**,** 5** with 2:1 and precursors** 8**,** 9** with 1:1 stoichiometric ratio between Li+ and Co2+.** Table S3.** Results of the argentometric titration of chloride and ICP-measurements for lithium.** Table S4.** ICP analysis for Li+ and Co3+ of LiCoO2 obtained from different precursors.** Figure S6.** XRD study of commercial LCO, and nano-LCO obtained from LiOtBu before annealing and after annealing at 600°C and 700°C.** Figure S7.** XRD of LiCoO2 from 9-LiOPh calcined at 450°C before washing. The red line corresponds to HT-LCO and the blue lines are Li2CO3.** Table S5.** The combustion temperature and the thermal measurement conditions of the compounds** 1**,** 8**-**12**.** Table S6.** TGA weight loss in percentage [%] with associated steps of compounds** 1**,** 8**-**12**.** Equation S1**-**S5.** Determination of the particle and crystallite sizes.** Figure S8.** Morphologies of LiCoO2 prepared with different precursors at 450°C.** Figure S9.** (a) Cyclic voltammograms of the 15 nm LCO prepared from the compound 12 at different sweep rates. (b) The maximum anodic and cathodic current peaks of LiCoO2 electrode versus the square root of sweep rate.** Table S7.** Li+ diffusion coefficients determined for HT-LCO obtained from different precursors.** Figure S10.** Nyquist plot for LiCoO2 electrodes from LiOtBu with fit: filled markers – experimental points, open markers – fit points with error bars a) and corresponding equivalent circuit model b) with fitting report c).** Figure S11.** Nyquist plot obtained for LiCoO2 electrodes from LiOPh with fit: filled markers – experimental points, open markers – fit points with error bars a) and corresponding equivalent circuit model b) with fitting report c).

## Abbreviations

HT-LCO: high temperature LiCoO_2_
LT-LCO: low temperature LiCoO_2_
THF: tetrahydrofuran
ALI: air-liquid interface
LiOPh: lithium phenoxide
LiO^*t*^Bu: lithium *tert*-butoxide
LiOMe: lithium methoxide
LiOEt: lithium ethoxide
LiO^*i*^Pr: lithium *iso*-propoxide
TMEDA: tetramethylethylenediamine
DME: dimethoxyethane
Py: pyridine
IR: infra-red
NMR: nuclear magnetic resonance
XRD: X-ray diffraction
*M*: molecular mass
*a, b, c*: unit cell dimensions
*β*: monoclinic angle
*V*: unit cell volume
Z: number of independent asymmetric units per unit cell
*ρ*_calcd_: density
*T*: temperature at which single crystals were measured
Θ: theta angle for single crystal x-ray measurements
*GOOF*: goodness of fit
*R*1, w*R*2: quality factors
TGA: thermogravimetric analysis
STDA: simultaneous differential thermal analysis
BET: Brunauer, Emmett and Teller
SEM: scanning electron miscroscope
FEI: field electron and ion
EDS: energy dispersive X-ray spectroscopy
ICP-OES: inductively coupled plasma optical emission spectrometry
PVDF: polyvinylidene fluoride
NMP: N-methyl-2-pyrrolidone
ABG and SFG: name of graphite provided from the manufacturer
EC: ethylene carbonate
DMC: dimethyl carbonate
DEC: diethylene carbonate
C/20, C/10, C/5, C/2, 1C and 20C: charging for 20 h, 10 h, 5 h, 2 h, 1 h and 3 mins
EIS: electrochemical impedance spectroscopy
FRA: frequency response analyser
RPMI: Roswell Park Memorial Institute
PET: polyethylene terephthalate
CH: cluster of differentiation
DP: dry powder insufflator
ELISA: enzyme-linked immunosorbent assay
BVS: bond-valence-sum
