# Supplementary material for: Characteristics and properties of nano-LiCoO2 synthesized by pre-organized single source precursors: Li-ion diffusivity, electrochemistry and biological assessment
Source: J Nanobiotechnology. 2017 Aug 22;15:58. doi: 10.1186/s12951-017-0292-3 (PMC5568213; doi:10.1186/s12951-017-0292-3)
Supplement: Supplementary file 1 — Additional file1: Text 1. Synthesis of bimetallic compounds. Table S1. Crystal data. Text 2. Single crystal structure descriptions. Text 3. Argentometric titration. Table S2. Idealistic oxidation reactions of two types of compounds, precursors 1, 5 with 2:1 and precursors 8, 9 with 1:1 stoichiometric ratio between Li+ and Co2+. Table S3. Results of the argentometric titration of chloride and ICP-measurements for lithium. Table S4. ICP analysis for Li+ and Co3+ of LiCoO2 obtained from different precursors. Figure S6. XRD study of commercial LCO, and nano-LCO obtained from LiOtBu before annealing and after annealing at 600°C and 700°C. Figure S7. XRD of LiCoO2 from 9-LiOPh calcined at 450°C before washing. The red line corresponds to HT-LCO and the blue lines are Li2CO3. Table S5. The combustion temperature and the thermal measurement conditions of the compounds 1, 8-12. Table S6. TGA weight loss in percentage [%] with associated steps of compounds 1, 8-12. Equation S1-S5. Determination of the particle and crystallite sizes. Figure S8. Morphologies of LiCoO2 prepared with different precursors at 450°C. Figure S9. (a) Cyclic voltammograms of the 15 nm LCO prepared from the compound 12 at different sweep rates. (b) The maximum anodic and cathodic current peaks of LiCoO2 electrode versus the square root of sweep rate. Table S7. Li+ diffusion coefficients determined for HT-LCO obtained from different precursors. Figure S10. Nyquist plot for LiCoO2 electrodes from LiOtBu with fit: filled markers – experimental points, open markers – fit points with error bars a) and corresponding equivalent circuit model b) with fitting report c). Figure S11. Nyquist plot obtained for LiCoO2 electrodes from LiOPh with fit: filled markers – experimental points, open markers – fit points with error bars a) and corresponding equivalent circuit model b) with fitting report c). [file 12951_2017_292_MOESM1_ESM.docx]

Additional file 1

**Characteristics and properties of nano-LiCoO_2_ synthesized by pre-organized single source precursors: Li-ion diffusivity, electrochemistry and biological assessment**

Jean-Pierre Brog^1^, Aurélien Crochet^2^, Joël Seydoux^1^, Martin J. D. Clift^3^, Sivarajakumar Maharajan^1^, Hana Barosova^3^, Pierre Brodard^4^, Mariana Spodaryk^5, 6^, Andreas Züttel^5, 6^, Barbara Rothen-Rutishauser^3^, Nam Hee Kwon^1^*, Katharina M. Fromm^1^*

^1^ University of Fribourg, Department of Chemistry, Chemin du Musée 9, CH-1700 Fribourg, Switzerland

^2^ University of Fribourg, Fribourg Center for Nanomaterials FriMat, Chemin du Musée 9, CH-1700 Fribourg, Switzerland

^3^ University of Fribourg, Adolphe Merkle Institute, CH-1700 Fribourg, Switzerland

^4^ University of Applied Sciences of Western Switzerland, College of Engineering and Architecture of Fribourg, Boulevard de Pérolles 80, CH-1705 Fribourg, Switzerland

^5^ École Polytechnique Fédérale de Lausanne (EPFL), Laboratory of Materials for Renewable Energy (LMER), ISIC-SB, Valais/Wallis Energypolis, Rue de l’Industrie 17, CH-1951 Sion, Switzerland

^6^ Empa Materials Science and Technology, Dübendorf, Switzerland

* Corresponding authors:

Dr. Nam Hee Kwon, University of Fribourg, Department of Chemistry, Chemin du Musée 9, CH-1700 Fribourg, Switzerland, Tel: +41 26 300 87 35, [namhee.kwon@unifr.ch](mailto:namhee.kwon@unifr.ch)

Prof. Dr. Katharina M. Fromm, University of Fribourg, Department of Chemistry, Chemin du Musée 9, CH-1700 Fribourg, Switzerland, Tel: +41 26 300 87 32, [katharina.fromm@unifr.ch](mailto:katharina.fromm@unifr.ch)

**Text 1**. Synthesis of bimetallic compounds.

**Table S1.** Crystal data.

**Text 2.** Single crystal structure descriptions.

**Text 3.** Argentometric titration.

**Table S2.** Idealistic oxidation reactions of two types of compounds, precursors **1**, **5**with 2:1 and precursors **8**, **9** with 1:1 stoichiometric ratio between Li^+^ and Co^2+^.

**Table S3.** Results of the argentometric titration of chloride and ICP-measurements for lithium.

**Table S4.** ICP analysis for Li^+^ and Co^3+^ of LiCoO_2_ obtained from different precursors.

**Figure S6.** XRD study of commercial LCO, and nano-LCO obtained from LiOtBu before annealing and after annealing at 600°C and 700°C.

**Figure S7.** XRD of LiCoO_2_ from 9-LiOPh calcined at 450°C before washing. The red line corresponds to HT-LCO and the blue lines are Li_2_CO_3_.

**Table S5.** The combustion temperature and the thermal measurement conditions of the compounds **1**, **8**-**12**.

**Table S6.** TGA weight loss in percentage [%] with associated steps of compounds **1**, **8**-**12**. **Equation S1-S5.** Determination of the particle and crystallite sizes.

**Figure S8.** Morphologies of LiCoO_2_ prepared with different precursors at 450°C.

**Figure S9.** (a) Cyclic voltammograms of the 15 nm LCO prepared from the compound 12 at different sweep rates. (b) The maximum anodic and cathodic current peaks of LiCoO_2_ electrode versus the square root of sweep rate.

**Table S7.** Li^+^ diffusion coefficients determined for HT-LCO obtained from different precursors.

**Figure S10.** Nyquist plot for LiCoO2 electrodes from LiOtBu with fit: filled markers – experimental points, open markers – fit points with error bars a) and corresponding equivalent circuit model b) with fitting report c).

**Figure S11.** Nyquist plot obtained for LiCoO_2_ electrodes from LiOPh with fit: filled markers – experimental points, open markers – fit points with error bars a) and corresponding equivalent circuit model b) with fitting report c).

**Text 1. Synthesis of bimetallic compounds**

**[Co(OPh)_4_Li_2_(THF)_4_] (1)** : CoCl_2_ (0.1 g, 0.77 mmol) was dried for 2 h under vacuum at 300°C and then dissolved in 10 ml of THF, heating to reflux under magnetic stirring during 30 min. 3.1 ml (3.1 mmol) of a solution of LiOPh 1.0 M in THF were added slowly and the mixture was heated to reflux under magnetic stirring during 30 min. The solution was concentrated, filtrated and 10 ml of heptane was added. The mixture was left at RT. Violet single-crystals of **1** suitable for X-ray analysis grew within some weeks with a yield of 82 % with respect to CoCl_2_. *IR: 3553 cm^-1^ (w), 3067(w), 2978(w), 2879(w), 1558(s),1481(s), 1283(s), 1246(s), 1165(s), 1068(m),1040(m), 995(sh), 874(sh), 841(m), 827(m), 754(s), 689(s),624(sh).* *1H NMR: (DMSO-d6, 360MHz): 6.98ppm (br, OPh), 3. 63 (br, THF OCH2),1.68 (br, THF CH2).*

**[Co(OPh)_4_Li_2_(THF)_4_]·THF (2) :** Compound **2** was obtained in the same way as **1**, except that the mixture was left at -24°C. Violet single-crystals of **2** suitable for X-ray analysis grow within some weeks with a yield of 56 % with respect to CoCl_2_. *IR: 3553 cm^-1^ (w), 3393(w), 3067(w), 2978(w), 2879(w), 1558(s),1481(s), 1283(s), 1246(s), 1165(s), 1068(m),1040(m), 995(sh), 874(sh), 841(m), 827(m), 754(s), 689(s),624(sh).*

**[Co(OPh)_4_Li_2_(THF)_2_(H_2_O)(THF)_2_]_2_ (3) :** Compound **3** was obtained under similar conditions as **1**, but crystallization took place at -24°C in air. Violet single-crystals of **3** suitable for X-ray analysis grow within some weeks, with a very low yield of less than 10 % (degradation of the solution due to the presence of water).

**[Co(OPh)_4_Li_2_(TMEDA)_2_] (4) :** CoCl_2_ (0.1 g, 0.77 mmol) was dried for 2 h under vacuum at 300°C and then dissolved in 10 ml of THF and heated to reflux under magnetic stirring during 30 min. Afterwards 3.1 ml (3.1 mmol) of a solution of LiOPh 1.0 M in THF was added slowly and the mixture was heated to reflux under magnetic stirring during 30 min. The mixture was evaporated to dryness and the residue was dissolved in 8 ml of TMEDA and heated to reflux for 1 h. The solution was concentrated, filtrated and the mixture was left at 4°C. Violet single-crystals of **4** suitable for X-ray analysis grew within some weeks with a yield of 69 % with respect to CoCl_2_. *IR: 2986 cm^-1^ (sh), 2956(w), 2832(w), 2790(w), 1587(m), 1486(s), 1463(s), 1354(sh), 1272(s), 1167(m), 1099(m), 1063(sh), 1036(sh), 1020(m), 992(m), 947(m), 842(m), 756(s), 690(s), 623(sh).*

**[Co(OPh)_4_Li_2_(dioxane)_2_]_n_ (5) :** Compound **5** was obtained under similar conditions as **4**, except that after evaporation to dryness, the residue was dissolved in 12 ml of dioxane and heated to reflux for 1 h. The solution was concentrated, filtrated and 3 ml of heptane were added, finally the mixture was left at RT. Violet single-crystals of **5** suitable for X-ray analysis grew within some weeks with a yield higher than 95 % with respect to CoCl_2_. *IR: 3374 cm^-1^ (w), 3062(sh), 3023(sh), 2971(sh), 2926(sh), 2865(sh), 1588(m), 1479(s), 1424(sh), 1374(sh), 1257(s), 1230(m), 1163(m), 1111(s), 1078(m), 1044(sh), 1214(sh), 892(sh), 869(m), 843(sh), 758(s), 694(s), 616(m)*

**[Co(OPh)_4_Li_2_(DME)_2_] (6) :** Compound **6** was obtained under similar conditions as **4**, except that the residue was dissolved in 12 ml of DME and heated to reflux for 1 h. The solution was concentrated, filtrated and 3 ml of heptane were added, finally the mixture was left at RT. Blue-violet single-crystals of **6** non-suitable for X-ray analysis grew in some weeks with a yield of 47 % with respect to CoCl_2_. *IR: 3397 cm^-1^ (w), 3061(sh), 3024(sh), 2938(w), 2833(sh), 1588(s), 1482(s), 1366(sh), 1278(s), 1192(sh), 1166(m), 1118(m), 1073(m), 1022(m), 995(m), 867(sh), 843(m), 756(s), 691(s), 623(m).*

**[Co(OPh)_4_Li_2_(Py)_4_] (7)** **:** Compound **7** was obtained under similar conditions as **4**, except that the residue was dissolved in 15 ml of Pyridine and heated to reflux for 1 h. The solution was concentrated, filtrated and 3 ml of heptane were added, finally the mixture was left at RT. Blue-violet single-crystals of **7** non-suitable for X-ray analysis grew within some weeks with a yield of 39% with respect to CoCl_2_. *IR: 3059 cm^-1^(sh), 3021(w), 3005(sh), 1604(sh), 1583(m), 1482(s), 1445(m), 1291(sh), 1291(s), 1238(sh), 1219(m), 1165(m), 1146(sh), 1070(m), 1041(m), 1013(m), 992(s), 946(sh), 874(m), 838(w), 754(s), 691(s), 633(m), 621(sh).*

**[Co_2_(O^t^Bu)_6_Li_4_(THF)_2_] (8) :** CoCl_2_ (585 mg, 4.5 mmol) was dried for 30 min under vacuum at 150°C and then dissolved in 10 ml of THF, heating to reflux under magnetic stirring during 15 min. 13.5 ml (13.5 mmol) of a solution of LiO*^t^*Bu 1 M in THF were added slowly and the mixture was heated to reflux under magnetic stirring during 30 min. The solution was concentrated and evaporated. Finally a polycristalline purple powder was obtained. Yield: 87 % with respect to CoCl_2_.  *IR: 3637 (sh), 3608 (sh), 3446 (w), 2960 (sh), 2923 (sh), 2854(sh), 1627(sh), 1465(m), 1463(m), 1382(sh), 1380(sh), 1244 (sh), 1196 (m), 1100 (w), 1030 (sh), 914(sh), 881(w), 838(sh), 663(m).*

**[Co_2_(O^t^Bu)_2_(OPh)_4_Li_2_(THF)_4_] (9) :** CoCl_2_ (500 mg, 3.85 mmol) was dried for 30 min under vacuum at 150°C and then dissolved in 10 ml of THF, heating to reflux under magnetic stirring during 15 min. 3.9 mL (3.9 mmol) of a solution of LiO*^t^*Bu 1 M in THF and 7.7 ml (7.7 mmol) of a solution of lithium phenoxide 1 M in THF were added slowly. Then, the mixture was heated to reflux under magnetic stirring during 30 min. The solution was concentrated and evaporated. Finally a polycristalline purple powder was obtained. Yield : 85 % with respect to CoCl_2_. *IR: 3060(m), 2968(m), 2878 (m), 1588 (sh), 1482 (sh), 1363 (m), 1276 (w), 1175 (s), 1165 (sh), 1068 (sh), 1045 (sh), 993 (sh), 947 (sh), 881 (m), 844(sh), 826 (sh), 756 (sh), 691 (sh), 623 (sh).*

**[Co_2_(O^i^Pr)_6_Li_2_(THF)_2_] (10) :** CoCl_2_ (500 mg, 3.85 mmol) was dried for 30 min under vacuum at 150°C and then dissolved in 10 ml of THF, heating to reflux under magnetic stirring during 15 min. 5.8 ml (11.6 mmol) of a solution of LiO*^i^*Pr 2 M in THF were added slowly and the mixture was heated to reflux under magnetic stirring during 30 min. The solution was concentrated and evaporated to dryness. A brown-black powder was obtained. Yield: 92 % with respect to CoCl_2_. *IR: 3060(m), 2968(m), 2878 (m), 1588 (sh), 1482 (sh), 1363 (m), 1276 (w), 1175 (s), 1165 (sh), 1068 (sh), 1045 (sh), 993 (sh), 947 (sh), 881 (m), 844(sh), 826 (sh), 756 (sh), 691 (sh), 623 (sh).*

**[Co_2_(OEt)_12_Li_8_(THF)_8-10_] (11) :** CoCl_2_ (500 mg, 3.85 mmol) was dried for 30 min under vacuum at 150°C and then dissolved in 10 ml of THF, heating to reflux under magnetic stirring during 15 min. 11.6 ml (23.2 mmol) of a solution of LiOEt 2 M in THF were added slowly and the mixture was heated to reflux under magnetic stirring during 30 min. The solution was concentrated and evaporated. Finally a glassy black powder was obtained. Yield: 89 % with respect to CoCl_2_. *IR: 3060(m), 2968(m), 2878 (m), 1588 (sh), 1482 (sh), 1363 (m), 1276 (w), 1175 (s), 1165 (sh), 1068 (sh), 1045 (sh), 993 (sh), 947 (sh), 881 (m), 844(sh), 826 (sh), 756 (sh), 691 (sh), 623 (sh).*

**[Co_2_(OMe)_6_Li_2_(THF)_2_(MeOH)_2_] (12) :** CoCl_2_ (500 mg, 3.85 mmol) was dried for 30 min under vacuum at 150°C and then dissolved in 10 ml of THF, heating to reflux under magnetic stirring during 15 min. 5.3 ml (11.7 mmol) of a solution of LiOMe 2.2 M in MeOH were added slowly and the mixture was heated to reflux under magnetic stirring during 30 min. The solution was concentrated and evaporated. Finally a yellow/sand colored powder was obtained. Yield: 90 % with respect to CoCl_2_. *IR: 3060(m), 2968(m), 2878 (m), 1588 (sh), 1482 (sh), 1363 (m), 1276 (w), 1175 (s), 1165 (sh), 1068 (sh), 1045 (sh), 993 (sh), 947 (sh), 881 (m), 844(sh), 826 (sh), 756 (sh), 691 (sh), 623 (sh).*

**Table S1. Crystal data**

|  | **1** | **2** | **3** | **4** | **5** | **9** |
| --- | --- | --- | --- | --- | --- | --- |
| Formula | C_40_H_52_CoLi_2_O_8_ | C_44_H_60_CoLi_2_O_9_ | C_64_H_76_Co_2_Li_4_O_14_, 4(C_4_H_8_O) | C_36_H_52_CoLi_2_N_2_O_4_ | C_36_H_44_CoLi_2_O_10_ | C_48_H_70_Co_2_Li_2_O_10_ |
| M_w_ / g.mol^-1^ | 733.66 | 805.8 | 1503.28 | 677.63 | 709.52 | 938.78 |
| T / K | 150(2) | 150(2) | 150(2) | 150(2) | 150(2) | 150(2) |
| Wavelength / Å | 0.71073 | 0.71073 | 0.71073 | 0.71073 | 0.71073 | 0.71073 |
| Crystal system | Monoclinic | Monoclinic | triclinic | Monoclinic | Monoclinic | triclinic |
| Space group | *P*2_1_/n | *P*2_1_/n | *P*-1 | *P*2_1_/n | *P*2_1_/n | *P*-1 |
| *a* / Å | 11.0605(6) | 20.7292(5) | 12.4747(9) | 14.5400(9) | 13.3438(4) | 9.3899(8) |
| *b* / Å | 26.1981(14) | 19.9745(7) | 12.7348(8) | 26.7380(9) | 18.3549(7) | 11.8195(10) |
| *c* / Å | 13.5136(7) | 10.7405(10) | 14.5658(9) | 20.8220(9) | 14.8540(4) | 13.6720(12) |
| *α* / ° | 90 | 90 | 74.925(5) | 90 | 90 | 106.442(7) |
| *β* / ° | 102.793(4) | 103.273(4) | 69.330(5) | 105.982(5) | 92.983(2) | 107.371(7) |
| *γ* / ° | 90 | 90 | 69.728(5) | 90 | 90 | 91.497(7) |
| *V* / Å3 | 3964.31(37) | 4328.37(42) | 2005.7(2) | 7721(7) | 3633.2(2) | 1378.8(2) |
| *Z* | 4 | 4 | 1 | 8 | 4 | 1 |
| *D*_c_ / g/cm3 | 1.23 | 1.24 | 1.245 | 1.157 | 1.297 | 1.131 |
| *F*(000) | 1555.8 | 1715.8 | 798 | 2888 | 1492 | 498 |
| θ range / ° | 1.5-25 | 1.4-19.5 | 1.5-26.7 | 1.3-22.7 | 1.8-23.6 | 1.64-23.62 |
| Refl. Coll./ Indep./ Obs | 6907/6907/1449 | 3635/3635/2084 | 8493/8493/1135 | 10378/10378/2883 | 5429/5429/1015 | 22629/4078/2287 |
| No. variables | 356 | 416 | 448 | 779 | 436 | 313 |
| GOOF (on *F*^2^) | 0.605 | 0.912 | 0.622 | 0.754 | 0.305 | 0.906 |
| *R*1 (I>2σ(I)) | 0.072 | 0.075 | 0.082 | 0.078 | 0.027 | 0.0612 |
| *wR*2 (all data) | 0.179 | 0.182 | 0.249 | 0.153 | 0.093 | 0.1396 |

For compounds **6-7**, the single crystal quality was not sufficient to solve the structure to satisfaction. Heavy atom positions indicate however a similar core as for compound **1**.

**Text 2. Single Crystal Structure Descriptions:**

In compounds **1** and **2**, [Co(OPh)_4_Li_2_(THF)_4_] (**1**) and [Co(OPh)_4_Li_2_(THF)_4_]·THF (**2**), a central Co^2+^ ion is coordinated tetrahedrally by four phenolate entities, bridging pairwise to two Li^+^ ions. The coordination spheres of the Li^+^ cations are completed by two THF molecules each (Figure 2). The Li–O(THF) distances for 1 (and 2) are respectively 1.94(1) Å (1.93(2) Å) for O5, 1.90(2) Å (1.94(2) Å) for O6, 1.93(2) Å (1.92(2) Å) for O7 and 1.96(2) Å (1.98(2) Å) for O8. The geometry of the Li^+^ cations is a tetrahedral with the angles O5–Li1–O6, O5–Li1–O1, O6–Li1–O2 of respectively 104.8(8)°, 113.6(8)°, 114.5(8)° for 1 and 104.1(8)°, 113.8(8)°, 112.8(8)° for 2, and for Li2, the angles O7–Li2–O8, O7–Li2–O3 and O8–Li2–O4 are 103.3(9)°, 111.1(9)° and 115.0(9)° for compound 1, and 104.7(9)°, 110.0(9)° and 114.8(9)° for compound 2. The Li^+^ cations in 1 are 2.80(2) Å (for Li1) and 2.79(2) Å (for Li2) apart from the central cobalt ion, while in 2, these values are 2.81(2) Å for Li1 and 2.82(2) Å for Li2.

The additional “free” THF molecule in compound 2 is connected to the Li_2_Co-moiety by a short contact between its O-atom O9 and H5 from the phenoxide group containing O1 (H5…O9 2.62(1) Å, C5–H5…O9 141.2(9)°) (Fig. S1). This has however only a weak influence on the O–M–O (M = Co, Li) angles.

**Figure S1.** Labelled view of the molecular structure of **2**, H-atoms are omitted for clarity

In compound 3, obtained by crystallization under non-inert conditions in THF, the structure is similar to 1 and 2, except that the terminal THF ligands of one of the two Li-ions, namely Li2, have been formally replaced by two water molecules (O7 and O7a), which act as bridging ligands between two [Li_2_Co(OPh)_4_] cores (Fig. S2). In addition, these two water molecules form hydrogen bonds via their H-atoms to four THF molecules (O8, O9 and their symmetry equivalents) with bond lengths of 2.67(1) Å for O7–O8 and 2.61(1) Å for O7–O9. The angle O8–O7–O9a is 103.2(4)°, with a = -x, -y, 2-z. The Li1 atom and its symmetry equivalent are tetracoordinated by oxygen atoms, two from the phenoxide and two from THF. These Li1–O distances are 1.93(2)Å for O1, 1.92(3)Å for O2, 2.00(2) Å for O5 and 1.88(2) Å for O6, and the angles O–Li1–O are 86(1)° for O1–Li1–O2, 122(1)° for O1–Li1–O6, 112(1)° for O1–Li1–O5 and 120(1)° for O5–Li1–O6. For Li2, coordinated by two phenoxide moieties and two water molecules, the Li2–O distances are 1.93(2) Å for O3, 1.95(2) Å for O4, 1.97(2) Å for O7 and 2.00(2) Å, while the O–Li2–O angles are 86(1)° for O3–Li2–O4, 124(2)° for O3–Li2–O7, 116(2)° for O4–Li2–O7 and 94(1)° for O7–Li2–O7a. The lithium cations and the cobalt cation are 2.82(2) Å and 2.86(3) Å away for Li1 and Li2, respectively. The water-bridged lithium cations are 2.70(4) Å apart from each other.

**Figure S2.** View of the molecular structure of **3**, H-atoms are omitted for clarity, a = -x, -y, 2-z

The recrystallization of **1** from TMEDA gives compound **4** in the monoclinic space group *P*2_1_/c. Contrary to **1** and **2**, the asymmetric unit of **4** contains two almost identical molecules A and A’, each forming a [Li_2_Co(OPh)_4_]-core similar as in **1**, but the coordination sphere of the lithium cations is completed by one TMEDA molecule (Fig. S3), formally replacing the two THF ligands. In molecule A, the bite angles of the N-atoms are 89.8(5)° for N1–Li1–N2 and 89.8(4)° for N3–Li2–N4, with N–Li distances of 2.05(1) Å for N1, 2.09(1) Å for N2, 2.08(1) Å for N3, and 2.074(9) Å for N4. In the second molecule A’, the bite angles of TMEDA with the lithium cations Li3 and Li4 are 88.3(5)° and 87.2(4)°, respectively, and the Li–N distances are 2.10 (1) Å for N5, 2.05(1) Å for N6, 2.09(1) Å for N7, 2.12(1) Å for N8. The lithium cations are 2.78(1) Å and 2.762(9) Å away from Co1, respectively 2.813(9) Å and 2.771(9) Å from Co2.

**Figure S3.** View of the molecular structure of **4**, H-atoms are omitted for clarity

Using a ligand with two donor-atoms like dioxane, which can only rather act as bridging ligand and not as chelating ligand, we obtained compound **5**, which crystallizes in the monoclinic space group *P*2_1_/n. Compared to compound **1**, all terminal THF ligands have been replaced by dioxane molecules (Fig. S4). One dioxane ligand on each Li^+^ ion acts as terminal ligand, while the other one forms a bridge towards the next neighbor [Li_2_Co(OPh)_4_] entity, yielding thus a 1D coordination polymer (Fig.S4). For Li1, the angles are 90.1(5)° for O1–Li1–O2, 116.3(6)° O1–Li1–O5, 119.2(6)° O1–Li1–O6 and 100.0(5)° for O5–Li1–O6; the distances Li1–O are 1.86(1) Å, 1.90(1) Å, 1.97(1) Å and 1.94(1) Å for O1, O2, O5 and O6, respectively. For Li2, the angles are 84.8(2)° for O3–Li2–O4, 113.4(6)° O3–Li2–O8, 120.4(6)° O3–Li2–O10 and 103.7(6)° for O5–Li2–O6; the distances Li2–O are 1.89(1) Å, 1.90(1) Å, 1.88(1) Å and 1.92(1) Å respectively for O3, O4, O8 and O10. The dioxane molecules adopt a chair conformation with torsion angles of -/+56.9(7)° and -/+56.9(7)° for the ones which act as bridging ligands, and 56.4(8)° and -60.1(7)° for dioxane containing O6 and O7, 57.5(7)° and -58.7(5)° for dioxane containing O8 and O9. The lithium-cobalt distances are 2.76(1)Å for Li1 and 2.79(1) for Li2.

**Figure S4.** View of the molecular structure of **5**, H-atoms are omitted for clarity

Using an O-donor similar to TMEDA like in **4**, namely DME, we obtained compound **6** in form of small needles. In analogy to **4**, we can propose the structure shown in Fig. 3 as the most probable one for compound **6**. We base this on the fact that compound **6** has similar spectroscopic features and is also very sensitive to air and decomposes easily. This indicates terminal ligation of DME rather than bridging ligands. In previous studies [34, 60, 61], we had shown that DME can very well act as terminal bidentate ligand on alkali and alkaline earth metal ions, but in some rare cases, DME was also observed to be a bridging ligand between two different metal ions, leading to coordination polymers [31]. Similarly, the THF molecules of **1** (and **2**) can formally also be replaced by pyridine moieties as in compound **7**, and a structure for this compound is proposed in Fig. S5 as well.

**Figure S5.** Proposed structure for compound **6** (left) and compound **7** (right)

The compound **9** crystallizes in the triclinic space group *P*-1. The compound forms molecules of [(thf)_2_Li(*μ*-OPh)_2_Co(*μ*-O*^t^*Bu)]_2_ in which the two O*^t^*Bu groups play the role of bridging ligands between the two Co^2+^ ions. An inversion center is found in the geometrical middle of the so formed Co_2_O_2_-ring. The OPh ligands bridge pairwise between the cobalt and lithium ions, while two THF molecules complete the coordination of the lithium ions (Fig. 5). The coordination sphere of the Co^2+^ cation can be described as a tetrahedron with four oxygen atoms of both phenoxide and *tert*-butoxide O1, O1’, O2 and O3 on each corner. The Co–O distances are almost identical with 1.95(1) Å for O1 and O1’ and 1.96(1) Å for O2 and O3. The angle O1–Co1–O2 is 124.4(2)°, 124.3(1)° for the O1–Co1–O3, 83.9(2)° for the O2–Co1–O3 angle and 80.2° for the O1–Co1–O1’. The Li1–O distances are 1.92(1) Å and 1.89(1) Å for O2 and O3, respectively. The Li–O distances for THF are 1.93(1) Å for O5 and 1.94(1) Å for O4, respectively. The geometry around the Li^+^ cations is tetrahedral. For Li1 this tetrahedron can be described by the O2–Li1–O3, O4–Li1–O5, O3–Li1–O4, O2–Li1–O5 angles which have values of 86.7(5)°, 104.3(5)°, 114.9(6)° and 114.7(6)°, respectively. The Li^+^ cations are at a distance of 2.84(1) Å from the Co^2+^ cation. The BVS values are 2.02 for Co1 and Co2, 1.18 for Li1 and Li2, indicating sufficient good coordination of the metal ions by their ligands, as it is also the case for compounds **1-5** (Table 6 in discussion).

**Text 3. Argentometric titration:**

The chloride titration was carried out using the Mohr’s method [1] with dichromate as indicator and using KH_2_PO_4_/NaOH as a pH buffer (pH=8) to avoid secondary reactions. Three correction tests and three blank tests (with a 0.01 M LiCl solution) were carried out to determine the offset between the end of the titration and the indicator color change.  Aqueous solution of 0.01 M of AgNO_3_, and 0.007 M of K_2_Cr_2_O_7_were used. 120 mg of precursors **8**, **10-12** were dissolved in 120 mL of deionized and carbonates free water. Then 40 ml of each of these solutions were titrated three times until the change of color of the indicator.

**Table S2.** Idealistic oxidation reactions of two types of compounds, precursors **1**, **5**with 2:1 and precursors **8**, **9** with 1:1 stoichiometric ratio between Li^+^ and Co^2+^

| **Compound** | **Oxydant** | **Final product** |
| --- | --- | --- |
| **Non-stoichiometric ratio : 2 Li : 1 Co** | | |
| **1** |  |  |
| **5** |  |  |
| **Stoichiometric ratio 1 Li :1 Co** | | |
| **8** |  |  |
| **9** |  |  |

**Table S3.** Results of the argentometric titration of chloride and ICP-measurements for lithium:

|  | ml AgNO_3_ | mol Cl^-^ | mol Li^+^ | Ratio Cl^-^/Li^+^ | Ideal ratio |
| --- | --- | --- | --- | --- | --- |
| O*^t^*Bu | 15.3 | 1.53E-04 | 9.07E-05 | 1.69 | 2.00 |
| O*^i^*Pr | 17.3 | 1.73E-04 | 1.00E-04 | 1.72 | 2.00 |
| OEt | 9.7 | 9.72E-05 | 2.20E-04 | 0.44 | 0.50 |
| OMe | 22.6 | 2.26E-04 | 1.27E-04 | 1.78 | 2.00 |

Offset (is included in the value above): -2.3 ml (delay of color changing)

The ratio Cl^-^/Li^+^ is below the theoretical value due to the presence of excess Li from LiCl remaining for the ICP measurements so there is an excess of Li.

**Table S4.** ICP analysis for Li^+^ and Co^3+^ of LiCoO_2_ obtained from different precursors

| LCO - precursor | element with wavelength | Quantity (ppm) | Li/Co ratio | RSD | Standard Error (SE) | plus SE | Minus SE |
| --- | --- | --- | --- | --- | --- | --- | --- |
| LiOMe 0x | Li 670,784 | 0.16 |  | 2 |  |  |  |
|  | Co 228,616 | 1.48 | 0.9 | 1 | 0.02 | 0.93 | 0.89 |
| LiOMe 1x | Li 670,784 | 0.3 |  | 2.4 |  |  |  |
|  | Co 228,616 | 2.75 | 0.9 | 0.66 | 0.02 | 0.95 | 0.91 |
| LiOEt 0x | Li 670,784 | 0.44 |  | 0.71 |  |  |  |
|  | Co 228,616 | 5.32 | 0.7 | 1.42 | 0.01 | 0.71 | 0.69 |
| LiOEt 1x | Li 670,784 | 0.4 |  | 0.23 |  |  |  |
|  | Co 228,616 | 3.95 | 0.9 | 0.48 | 0 | 0.87 | 0.86 |
| LiOEt 2x | Li 670,784 | 0.28 |  | 1.53 |  |  |  |
|  | Co 228,616 | 2.02 | 1.2 | 0.78 | 0.02 | 1.22 | 1.17 |
| LiO*^i^*Pr 0x | Li 670,784 | 0.34 |  | 0.23 |  |  |  |
|  | Co 228,616 | 3.23 | 0.9 | 0.62 | 0.01 | 0.91 | 0.9 |
| LiO*^i^*Pr 1x | Li 670,784 | 6.54 |  |  |  |  |  |
|  | Co 228,616 | 55.08 | 1 |  |  |  |  |
| LiO*^i^*Pr 2x | Li 670,784 | 9.07 |  |  |  |  |  |
|  | Co 228,616 | 72.95 | 1.05 |  |  |  |  |
| LiO*^t^*Bu 1x | Li 670,784 | 0.59 |  | 0.81 |  |  |  |
|  | Co 228,616 | 5.3 | 1 | 0.63 | 0.01 | 0.96 | 0.94 |
| LiO*^t^*Bu 2x | Li 670,784 | 0.54 |  | 0.88 |  |  |  |
|  | Co 228,616 | 4.52 | 1 | 0.65 | 0.01 | 1.02 | 1 |
| LiOPh 1x | Li 670,784 | 0.92 |  | 0.25 |  |  |  |
|  | Co 228,616 | 6.86 | 1.1 | 0.36 | 0 | 1.14 | 1.13 |
| LiOPh 2x | Li 670,784 | 0.5 |  | 1.43 |  |  |  |
|  | Co 228,616 | 4.05 | 1 | 0.5 | 0.02 | 1.06 | 1.03 |
| Mix 0x | Li 670,784 | 0.43 |  |  |  |  |  |
|  | Co 228,616 | 3.33 | 1.09 |  |  |  |  |
| Mix 1x | Li 670,784 | 6.91 |  |  |  |  |  |
|  | Co 228,616 | 52.36 | 1.11 |  |  |  |  |
| Mix 2x | Li 670,784 | 0.44 |  | 0.9 |  |  |  |
|  | Co 228,616 | 4.08 | 0.9 | 1.08 | 0.01 | 0.93 | 0.91 |
| Commercial HT-LCO | Li 670,784 | 0.2 |  |  |  |  |  |
|  | Co 228,616 | 1.65 | 1 |  |  |  |  |
| Ball milled LCO | Li 670,784 | 0.31 |  |  |  |  |  |
|  | Co 228,616 | 2.92 | 0.9 |  |  |  |  |

0x, 1x, and 2x correspond to the numbers of washing steps after calcination.

**X-ray powder diffractograms**

**Figure S6.** XRD study of commercial LCO, and nano-LCO obtained from LiO*^t^*Bu before annealing and after annealing at 600°C and 700°C

**Figure S7.** XRD of LiCoO_2_ from **9**-LiOPh calcined at 450^o^C before washing. The red line corresponds to HT-LCO and the blue lines are Li_2_CO_3_.

**Thermal decomposition to LiCoO_2_**

**Table S5.** The combustion temperature and the thermal measurement conditions of the compounds **1, 8-12.**

| **#** | **Process** | **Calcination nominal temp** | **Heating ramp [°C/min]** | **Nominal temperature time** | **Air flow** | **Cooling** | **Washing** |
| --- | --- | --- | --- | --- | --- | --- | --- |
| 1 | Oxidation of **1**, **8-12** | 300°C | 18 | 1h | 8 l/min | 5 min in the air  (temp max/5 min) | 3x water 50 ml 2x ethanol  for 100 mg  + centrifugation 5000 rpm 3 min |
| 2 | Oxidation of **1**, **8-12** | 450°C | 18 | 1 h |  |  |  |
| 3 | Oxidation of **1**, **8-12** | 500°C | 18 | 2h |  |  |  |
| 4 | Annealing of process #**3** | 600°C | 17 | 1h20 |  |  |  |
| 5 | Annealing of process #**4** | 700°C | 15 | 0.5 h |  |  |  |

**Table S6.** TGA weight loss in percentage [%] with associated steps of compounds **1, 8-12**.
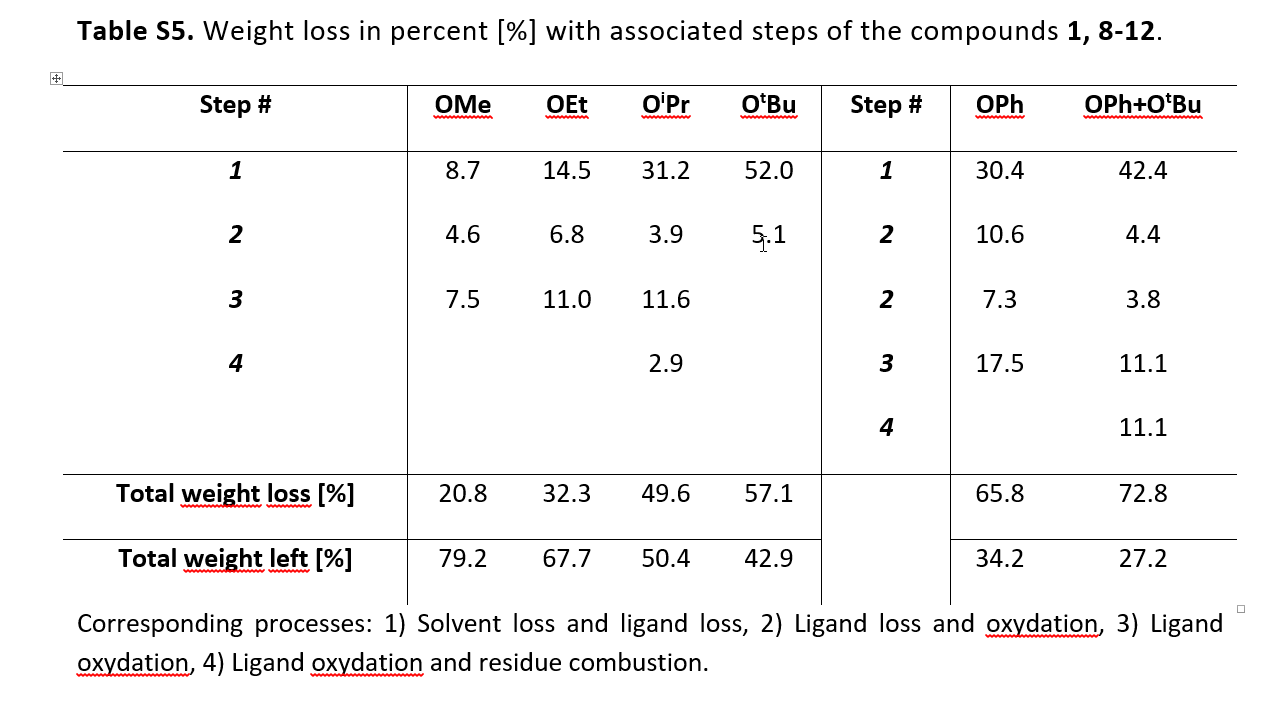


Step 1 : Solvent and ligand loss

Step 2 :Ligand loss and oxidation

Step 3: Ligand combustion and oxidation

Step 4: Ligand oxidation and organic residue combustion

The first major mass losses are associated with ligand losses: hydrolysis and evaporation. The second major mass losses are associated with ligands oxidation and decomposition.

Small minor steps are associated with either the elimination of organic residue at high temperature or evaporation/ligand loss steps at low temperature.

Some TGA steps are overlapped and cannot be well distinguished because of the heat rate needed to avoid the decomposition of the complex at the beginning of the experiment. Some TGA steps could be better pronounced under an inert atmosphere (N_2_) with a cap protection on the TGA crucibles. However, this kind of TGA does not represent the “real” decomposition in a large oven with air flow.

**Determination of the particle and crystallite sizes:**

The particle and crystallite size were assessed via the Scherrer equation (X-ray) and the BET equation (gas adsorption). The Scherrer equation [2, 3] links the broadening of the peaks in powder X-ray diffraction to the crystallite size.

$\tau=\frac{K\lambda}{\beta\cos(\theta)}$ (**Equation S1**)

With $\tau$the average size of the crystallite, K the shape factor, $\lambda$ the X-ray wavelength used for the measurement ,$\theta$the bragg angle of the diffraction plane considered and β the full width at half maximum (FWHM). The β parameter is the difference between the FWHM of a standard and the sample considered. It takes then instrumental effects into consideration.

$\beta= \beta_{obs.}- \beta_{std.}$ (**Equation S2**)

For the determination of the average particles size the BET gaz adsorption method was used.

By using the BET equation [4] :

$S_{w}=\frac{N\sigma V_{m}}{M_{v}}$ (**Equation S3**)

With S_w_ the specific surface area, N the Avogadro’s number, $\sigma$ the area occupied by one adsorbate molecule and M_v_ the gram molecular volume. For nitrogen as adsorbate gas at 77,36 K (liquid nitrogen temperature), this equation becomes

$S_{w}=4.35V_{m}$ (**Equation S4**)

With $V_{m}$ the volume of layers of gas (N_2_) adsorbed on the substrate.

From the specific surface area we can determine the average particle size:

$D= \frac{H x {10}^{4}}{\rho S_{w}}$ (**Equation S5**)

With H the shape factor (4 for spherial paritcles) and $\rho$the density of material (5.05 g/cc).

**TEM and SEM micrographs**

| **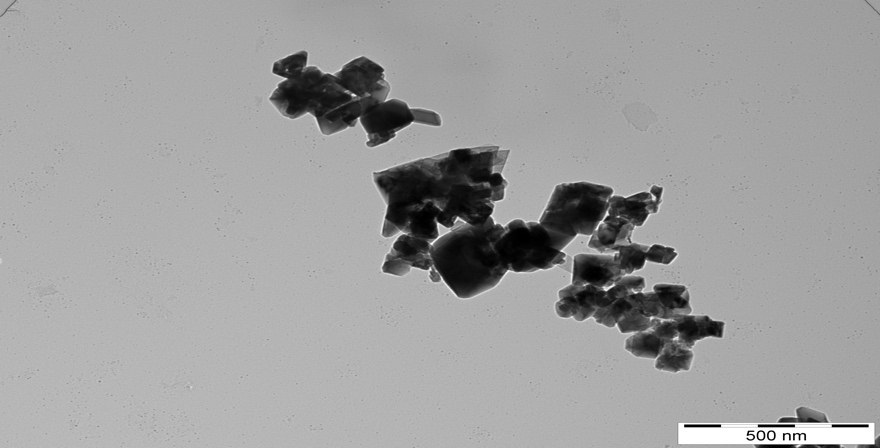** | | **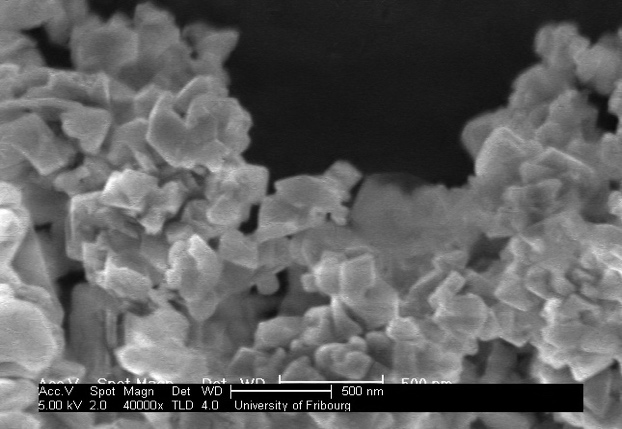** | |
| --- | --- | --- | --- |
| **Compound Phenoxide** | | | |
| **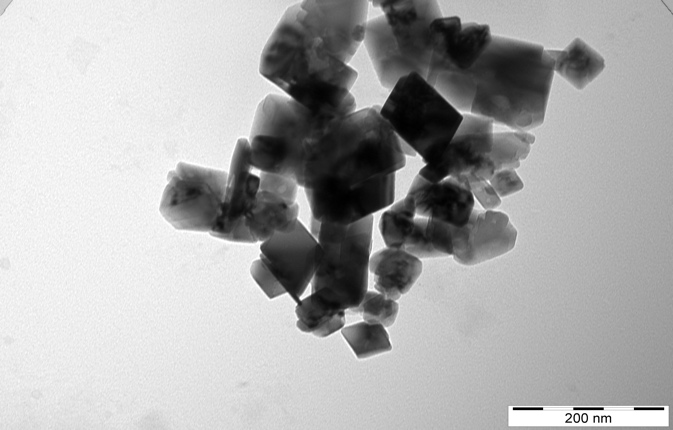** | | 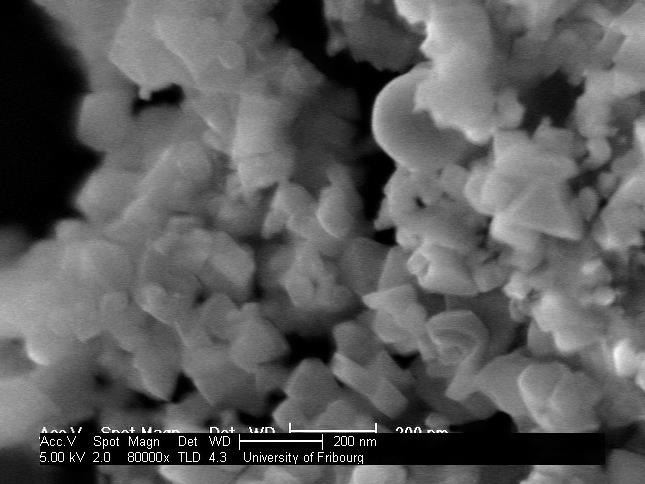 | |
| **Mix** | | | |
| **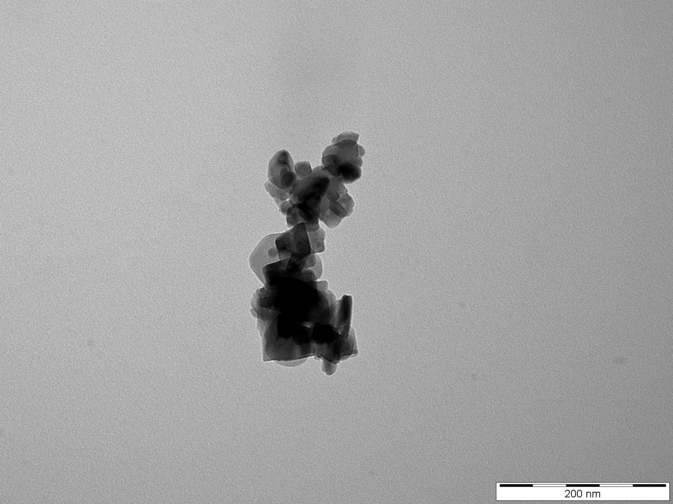** | | | 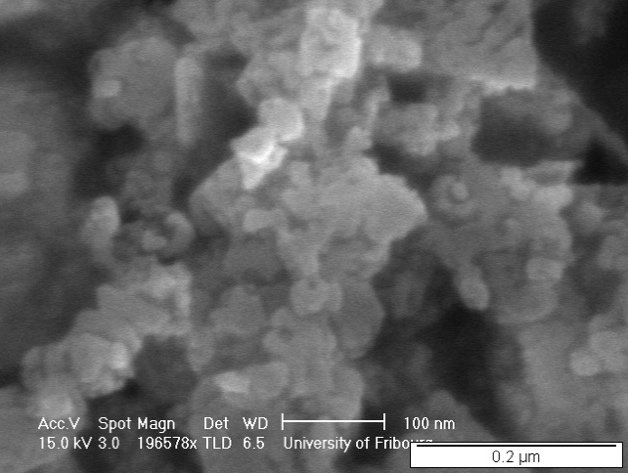 |
| ***Tert*-butoxide** | | | |
| **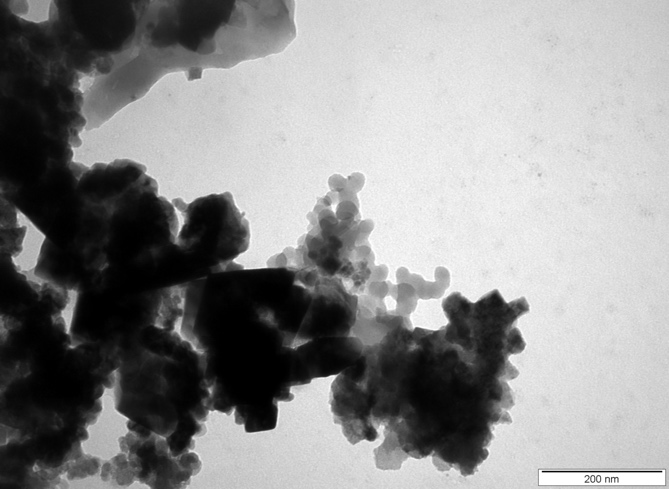** | | | 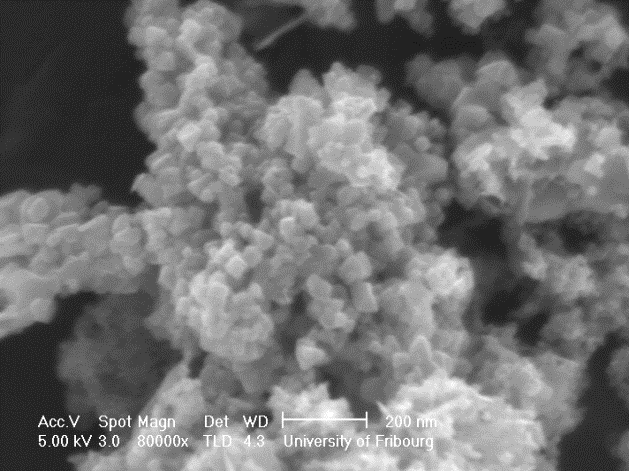 |
| **Isopropoxide** | | | |
| **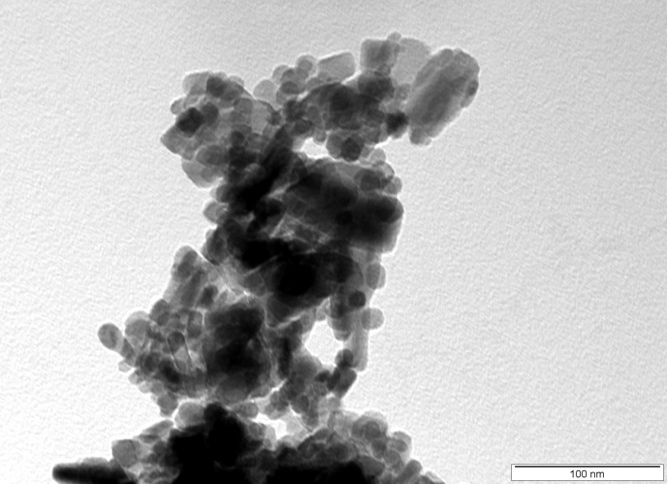** | 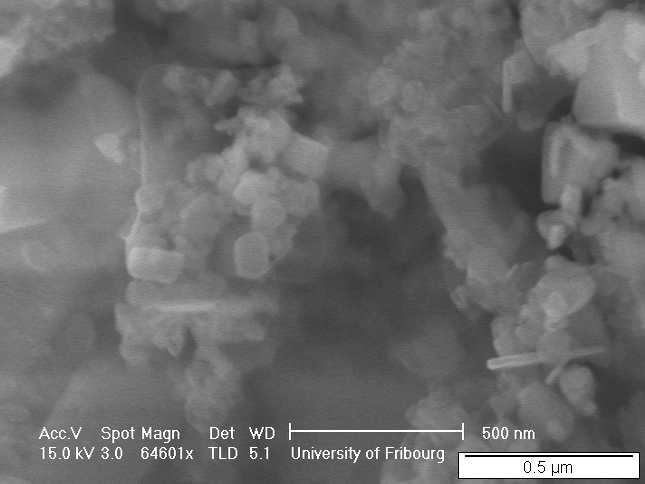 | | |
| **Ethoxide** | | | |
| **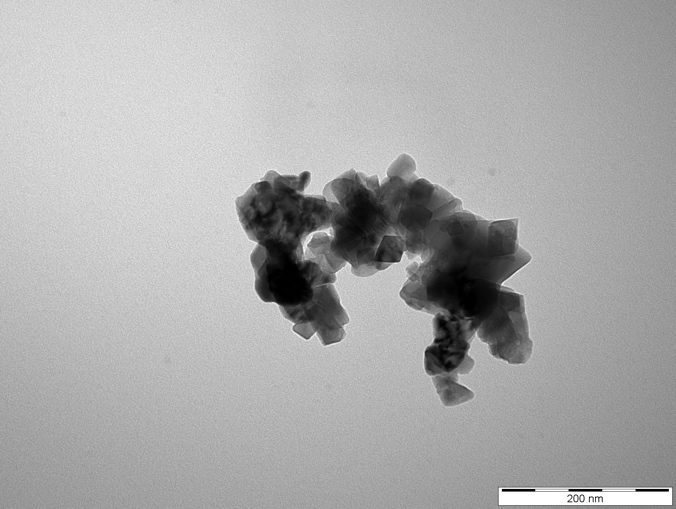** | | 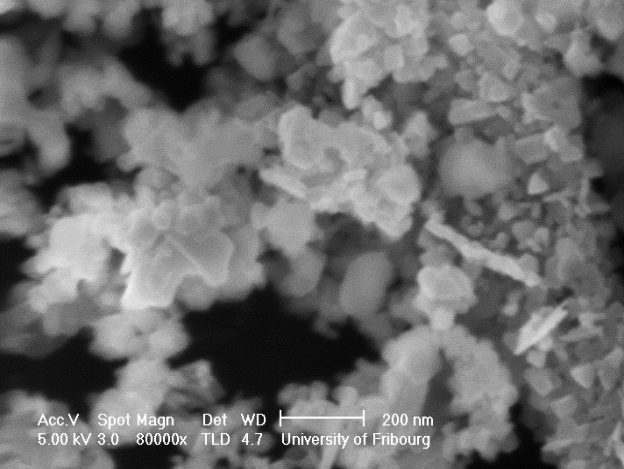 | |
| **Methoxide** | | | |

**Figure S8.** Morphologies of LiCoO_2_ prepared with different precursors at 450^o^C.

**Li^+^ diffusion coefficients of LiCoO_2_ electrodes.**

**Figure S9.** (a) Cyclic voltammograms of the 15 nm LCO prepared from the compound 12 at different sweep rates. (b) The maximum anodic and cathodic current peaks of LiCoO_2_ electrode versus the square root of sweep rate.

LiCoO_2_ prepared with different precursors were ball milled with carbon to make a composite for only 15 mins instead of 1 h. As shown in the table S4 below, the diffusion coefficients were not much different among the samples from different precursors. The short ball milling time (15 mins) may not be sufficient to make homogeneous composite between LiCoO_2_ and carbon.

**Table S7.** Li^+^ diffusion coefficients determined for HT-LCO obtained from different precursors

| LiCoO_2_-Precursor | D_Li_ (cm^2^/s) |
| --- | --- |
| **1**-LiOPh | 2.3 $\pm$ 1.83 E^-9^ |
| **8**-LiO*^t^*Bu | 6.6 $\pm$ 4.6 E^-10^ |
| **10**-LiO*^i^*Pr | 3.9 $\pm$ 3.3 E^-10^ |
| **12**-LiOMe | 2.2 $\pm$ 2.28 E^-10^ |

**EIS of LiCoO_2_ obtained from LiO*^t^*Bu**

In Fig. S10, the first resistor in the circuit is corresponding to the contact resistance and resistance of electrolyte. The small and big semicircles are represented by resistor and imperfect capacitor, or constant phase element (CPE).


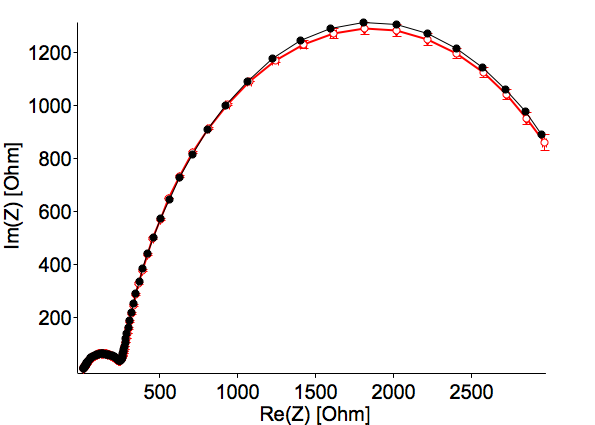

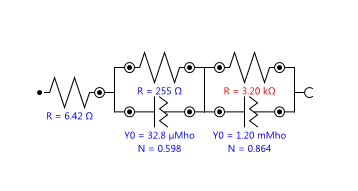


a) b)


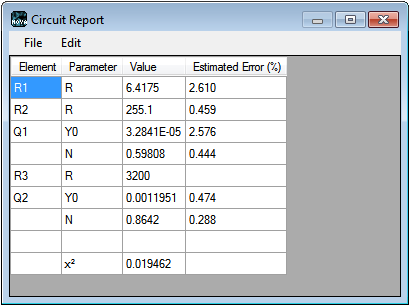


c)

**Figure S10.** Nyquist plot for LiCoO_2_ electrodes from LiO*^t^*Bu with fit: filled markers – experimental points, open markers – fit points with error bars a) and corresponding equivalent circuit model b) with fitting report c)**.**


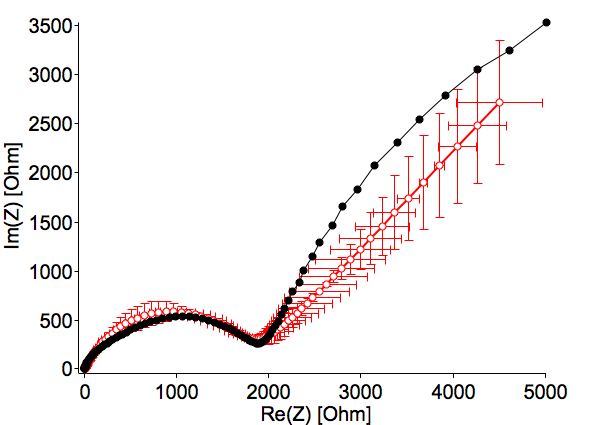

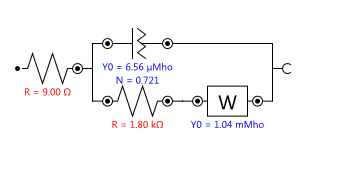


a) b)


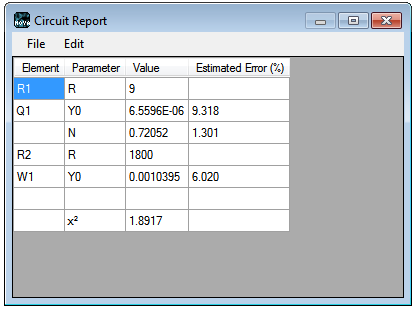


c)

**Figure S11.** Nyquist plot obtained for LiCoO_2_ electrodes from LiOPh with fit: filled markers – experimental points, open markers – fit points with error bars a) and corresponding equivalent circuit model b) with fitting report c)**.**

**References**

1. Doughty HW. Mother's method for the determination of silver and halogens in other than neutral solutions. J Am Chem Soc. 1924;46(12):2707–9. doi:DOI: 10.1021/ja01677a014.

2. Patterson AL. The Scherrer Formula for X-Ray Particle Size Determination. Physical Review. 1939;56(10):978-82. doi:10.1103/PhysRev.56.978.

3. Scherrer P. Bestimmung der Grosse und der Inneren Struktur von Kolloidteilchen Mittels Rontgenstrahlen. Göttinger Nachrichten Gesell. 1918;2:98-100.

4. Brunauer S, Emmett PH, Teller E. Adsorption of Gases in Multimolecular Layers. J Am Chem Soc. 1938;60(2):309-19.
